# Antimicrobial and Cytotoxic Activity of Novel Imidazolium-Based Ionic Liquids

**DOI:** 10.3390/molecules27061974

**Published:** 2022-03-18

**Authors:** Łukasz Pałkowski, Maciej Karolak, Andrzej Skrzypczak, Marta Wojcieszak, Filip Walkiewicz, Jonasz Podemski, Karol Jaroch, Barbara Bojko, Katarzyna Materna, Jerzy Krysiński

**Affiliations:** 1 Department of Pharmaceutical Technology, Faculty of Pharmacy, Nicolaus Copernicus University, Jurasza 2, 85-089 Bydgoszcz, Poland; maciej.karolak@cm.umk.pl (M.K.); jerzy.krysinski@cm.umk.pl (J.K.); 2 Faculty of Chemical Technology, Poznan University of Technology, Berdychowo 4, 60-965 Poznan, Poland; andrzej.skrzypczak@put.poznan.pl (A.S.); marta.d.wojcieszak@doctorate.put.poznan.pl (M.W.); filip.walkiewicz@put.poznan.pl (F.W.); katarzyna.materna@put.poznan.pl (K.M.); 3 Department of Pharmacodynamics and Molecular Pharmacology, Faculty of Pharmacy, Nicolaus Copernicus University, Jurasza 2, 85-089 Bydgoszcz, Poland; j.podemski@cm.umk.pl (J.P.); karol.jaroch@cm.umk.pl (K.J.); bbojko@cm.umk.pl (B.B.)

**Keywords:** ionic liquids, imidazolium compounds, antimicrobial properties, cytotoxicity

## Abstract

In this study, a series of 10 novel 1-methyl-3-octyloxymethylimidazolium derivatives carrying various anionic moieties (4-hydroxybenzenesulfonate, benzenesulfonate, carvacroloxyacetate, chloride, formate, propionate, thymoloxyacetate, vanillinoxyacetate, eugenoloxyacetate and trimethylacetate) were synthesized. Compounds were tested for their antimicrobial activity against six microbe strains (Staph-ylococcus aureus, Pseudomonas aeruginosa, Klebsiella pneumoniae, Escherichia coli, Enterococcus faecalis, and Candida albicans), cytotoxic activity against the mouse melanoma cell line (B16 F10), and surface active properties. All synthesized compounds exhibited antimicrobial activity (expressed as minimum inhibitory concentration; in range of 0.10–27.82 mM/L), especially against Gram-positive bacteria and fungi. In addition, all compounds demonstrated cytotoxicity on B16 F10 cells (IC50 values 0.0101–0.0197 mM/L). Surface properties defined as CMC values, ranged from 0.72 to 32.35 mmol L-1. The obtained results provide an insight into the promising activity of a novel group of quaternary imidazolium derivatives having ionic liquid properties. The most potent compounds, containing a thymoloxyacetate and eugenoloxyacetate moiety, could be candidates for new antimicrobial agents or surfactants.

## 1. Introduction

Ionic liquids (ILs) are a class of salts with melting points lower than 150 °C, and a large part of this class is liquid at room temperature. Due to their very diverse and unique physico-chemical properties, in recent years, these compounds have been seen as very promising alternatives to conventional organic solvents. Their features include their low vapor pressure, low flammability, and high thermal stability, so they have been labeled as environmentally friendly. However, the most important advantage of these compounds is the possibility of adjusting them to the needs of a specific chemical reaction or other processes [1,2,3,4].

The presence of ILs in the liquid state at relatively low temperatures is due to the presence of structures hindering crystallization. Among them, one can distinguish hydrogen bonds between the cation and the anion, as well as the considerable size and strong asymmetry of the organic cation. On the basis of the positive charge location, a division into ammonium, phosphonium, sulfonium, and oxonium ionic liquids can be determined. To date, the best described are the ammonium ILs, while the least known are the oxoniums, many of which are metastable.

Ionic liquids are widely used in pharmaceuticals. The most important areas of application include using ILS as biologically active compounds (drugs or potential medicinal products); synthesis of active substances (catalysts, chemical reaction media, biotechnology); optimization of the bioavailability of medicinal products (elimination of polymorphism, improvement of the solubility of active substances (combinations of IL with known drugs), carriers of medicinal substances); and analysis of medicinal products (electrophoresis, chromatography, extraction) [5,6,7].

Some ILs are biologically active compounds. The antimicrobial properties of quaternary halogenated ammonium derivatives are well known. At low concentrations, these compounds have a biostatic effect on most bacteria, mycobacteria and spores, fungi, and algae, while at average concentrations they act as biocidals against bacteria, fungi, algae, and lipophilic viruses [8,9].

ILs have proven, so far, to be active against Staphylococcus aureus, Enterococcus faecium, Escherichia coli, Micrococcus luteus, Staphylococcus epidermidis, Klebsiella pneumoniae, Candida albicans, Roseomonas rubra, and Streptococcus mutans, without toxic effects (at therapeutic concentrations) on mammalian cells. Especially, 1-alkyl-3-methylimidazolium is characterized by a high efficiency. They are derivatives of natural L-amino acids with a targeted activity against clinically significant microbial pathogens, including methicillin-resistant Staphylococcus aureus. This is of particular importance, taking into account the fact that these microorganisms can also be included in a biofilm, which prevents the penetration and action of antibiotics or disinfectants [10,11,12].

In the search for active derivatives targeted at specific biological goals, the adaptability of the physical, chemical, and biological properties of ILs, by building molecules with specific properties, and resulting from the selection of an appropriate cation and/or anion, has proven to be very helpful. Observing the antiseptic activity of ILs, some relationships were established, including the fact that their properties are determined to a greater extent by the nature of the cation structure than the anion structure. It has also been shown that ILs with a 1-alkyl-3-methylimidazolium cation are more active than those that are built based on a phosphonium or ammonium cation. Moreover, since the potency of the antimicrobial effect increases with the growth in hydrophobicity of ILs, the length of the alkyl substituent of the cation is an important structural element. The optimal structure contains 10 to 16 carbon atoms for alkyl, or 8 to 14 carbon atoms for alkoxymethyl groups [13,14].

In recent years, our team has studied the synthesis, biological and surface activity, and structure–activity relationships (SAR) of quaternary ammonium salts. In the scope of our interest are mainly mono- and bis-imidazolium salts [15,16,17]. We found that the following structures of bis-imidazolium chlorides have the optimal antimicrobial activity: 3,3’- (α, ω-dioxaalkane) bis (1-alkylimidazolium chloride), which has at least six carbon atoms in its linker, and has a R substituent of C7 to C11; α, ω-bis [(alkane) imidazol-1-yl-3-alkoxymethyl] chloride, which has at least five carbon atoms in the linker, and has a C7 to C13; and 3,3’- (α, ω-dioxaalkane) bis (1-alkylimidazolium) chloride or α, ω-bis [(alkane) imidazol-1-yl-3- alkoxymethyl chloride], which has at least seven carbon atoms and has a substituent R of C7 to C11. Moreover, we proved that surface parameters also have a prognostic value. Active antimicrobial bis-imidazolium chloride compounds possess −logCMC from 2.51 to 2.86 and surface surplus Γ in the range of 2.07–2.47 mol/m^2^.

Encouraged by the substantial antimicrobial activity of a few derivatives, especially against Gram-positive bacteria, we decided to extend our studies to new compounds, with a modified structure of anions. We obtained a novel group of imidazolium based compounds, which can be classified as ionic liquids. In this work, for the first time, we publish a description of the synthesis of this group of compounds and our research related to their antimicrobial and cytotoxic activity. Taking into account the growing resistance of bacteria to the drugs used in clinical practice, the search for new chemical compounds with antimicrobial activity is always relevant and necessary. The new compounds we have obtained are an important step in this direction.

## 2. Results and Discussion

**Figure 1:**
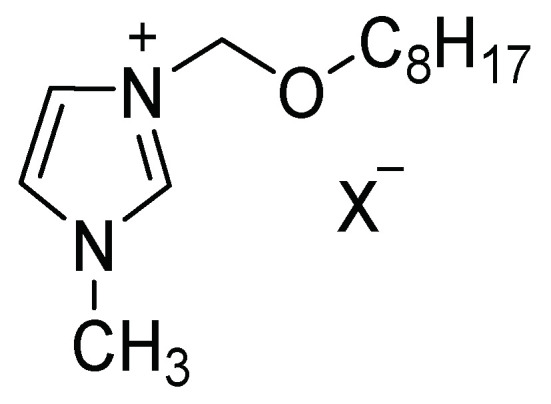
General Structure of New ILs.

In this paper we describe the synthesis and antimicrobial, cytotoxic, and surface activity of 10 novel ILs containing a cationic 1-methyl-3-octyloxymethylimidazolium moiety and different anions. The general structure can be found in Figure 1.

In Figure 1, X^−^ represents the following anions: 4-hydroxybenzenesulfonate (**FE-1**), benzenesulfonate (**BE-2**), carvacroloxyacetate (**OR-3**), chloride (**CL-4**), formate (**MR-5**), propioniate (**PR-6**), thymoloxyacetate (**TY-7**), vanillinoxyacetate (**VA-8**), eugenoloxyacetate (**EV-9**), and trimethylacetate (**TR-10**). The structures of anions are presented in Table 1.

### 2.1. Thermal Properties

#### 2.1.1. Phase Transition

The phase transition temperatures for the prepared ionic liquids are presented in Table 2 and the Supplementary Material. As can be seen, all compounds melt at a temperature bellow 100 °C. All of them are liquid at room temperature. Some of prepared ionic liquids exhibited a melting point at very low temperatures, around −40 °C, such OR-3 and PR-6 and EV-9, or even −61 °C for salt with a trimethylacetate anion (TR-10). The isomers-thymoloxyacetate (TY-7) and carvacroloxyacetate (OR-3) exhibited very similar glass transition temperatures, at around −70 °C, but only in cases where carvacroloxyacetate anion crystallization and melting point were observed. Cases with sulfate (FE-1 and BE-2) incorporation of a hydroxyl substituent into the benzene ring in para- position caused a reduction in glass transition, and in crystallization and melting point temperatures.

#### 2.1.2. Thermal Stability

Thermal stabilities of studied ILs are summarized in Table 3 and in the Supplementary Materials. Sulfates (FE-1 and BE-2) are stable up to 135–138 °C. The second group are phenoxyacetates (carvacroloxy-OR-3, thymoloxy-TY-7, vanilinoxy-VA-8, and eugenoloxyacetate EV-9), which start to decompose at 112 °C. The lower thermal stability was characterized for carboxylates, such as formate MR-5, propionate –PR-6, and purivate TR-10; these group start to decompose at 85 °C, as for propionate.

#### 2.1.3. Infrared Spectra Analysis

**Figure 2:**
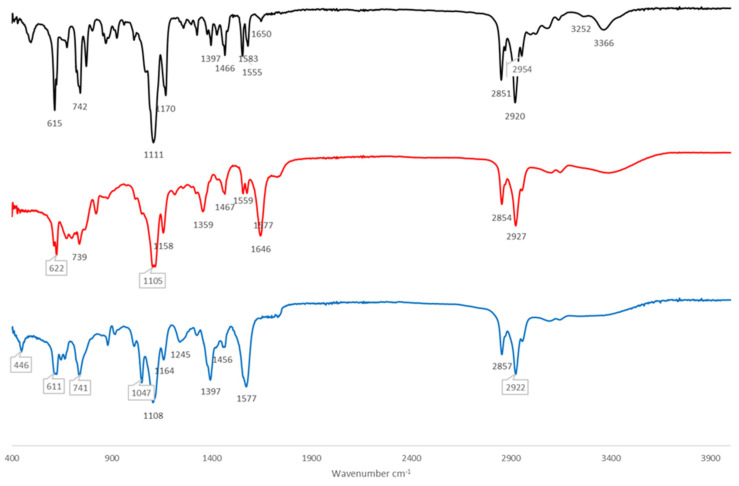
FTIR ATR spectra for CL-4 (black), MR-5 (red), and PR-6 (blue).

The ATRFT-IR spectra of the prepared ionic liquids are presented in Figure 2 and in the Supplementary Materials. As can be seen from the spectra, the bands in all of the catalysts within the range from 4000 to 500 cm^−1^ are similar, indicating that they have a similar structure. The bands at 3130 and 3082 cm^−1^ come from the stretch vibration of heterocyclic C-H. The adsorption at around 2920 and 2856 cm^−1^ are assigned to the stretch vibration of C-H of substituent on the imidazole ring. The characteristic peaks at around 1570 and 1444 cm^−1^ can be attributed to the stretch vibration of C=N on the imidazole ring. The characteristic peak at 1488 cm^−1^ is attributed to the bending vibration of C-H. The strong peak at 1111 cm^−1^ belongs to the stretch vibration of the imidazole ring.

The characteristic peak for the ether system appears between 1130 and 1000 cm^−1^, and is more intensive in case of thymoloxy- and carvacroloxyacetates, with incorporation of another ether group in the case of vanilinoxy and eugenoloxyacetate causing further intensification of the peak. For unsaturated substituent in eugenoloxyacetate a peak appears at 1609 and for aldehyde substituent at 1671 cm^−1^, for sulfacids at 1033 and 998 cm^−1^, and hydroxy derivative at 1006 and 1027 cm^−1^.

### 2.2. Antimicrobial Activity

The mechanism of the antiseptic action of ILs is closely related to their chemical structure and physical properties. ILs cations (especially heterocyclic) interact electrostatically with the negatively charged cell surfaces of microbes, and surface active compounds easily penetrate through the protein–lipid biological membranes, causing disturbances in their structural and functional coherence. Denaturation of bacterial proteins, disruption of complexes of nucleoprotein, and reactions with amino groups, consequently, lead to permanent damage and death of pathogen cells [6,18].

The antimicrobial properties of ILs are also related to the chain length of hydrophobic surfactants; the longer the hydrophobic chain, the better the performance. However, they cannot be extended indefinitely, due to the non-linear, but parabolic course of the dependence curve. A decrease in the MIC value was shown with elongating substituents, while the type of linker between these chains (-NH, -O, -S), and nitrogen atoms was not significant for activity against *Staphylococcus aureus*. However, the type of linker had an influence on the selectivity of the tested compounds; whereby the amide group influenced a broader spectrum of activity against Gram-positive and Gram-negative bacteria and fungi. The most active compounds were found among surfactants containing from 10 to 12 carbon atoms in the hydrophobic chain. However, the antimicrobial activity decreases as the length of the alkyl chain increases above 12 (C14–C16) and shortens below 10 carbon atoms. The bactericidal properties of ILS are also influenced by their lipophilicity [19,20,21].

The antimicrobial activity of the synthesized compounds is presented in Table 4. At the end of the table, the MIC values of reference substances commonly used in disinfection and antiseptics, didecyldimethylammonium chloride (**ST-1**) and benzalkonium chloride (**ST-2**), are given. Basically, the bacteriostatic activity of the novel imidazolium-based ionic liquids depends on the species of the strains and the structure of compounds. The highest values of MIC, which indicate the lowest antimicrobial activity, were obtained for Gram-negative bacteria, *Pseudomonas aeruginosa* (PAE). The lowest values of MIC were obtained for Gram-positive staphylococci (SAU), and *Candida* yeasts (CAL). This phenomenon is widely described in literature and can be easily explained by differences in bacterial cell structure. Gram-negative bacteria possesses an outer membrane of the cell wall, which limits the penetration of biocides into the cell. The activity of QASs is related to interruption of the cell wall integrity, and the membrane provides an additional protection for the cell [22]. Referring to the structure of the anion, TY-7 exhibited the highest antimicrobial activity against almost all tested strains, with the best results for *Staphylococcus aureus* and *Enterococcus faecalis* (EFA). On the other hand FE-1 exhibited the lowest biological activity and cannot be considered a potential antimicrobial agent. There is no clear explanation, since the structure of the anions FE-1 and BE-2 are similar and its antimicrobial activity is different. This may be related to the presence of a hydroxyl group in the structure, which is not present in the other tested anionic moieties. It is worth emphasizing that most of the tested compounds presented a bacteriostatic activity, and this depended on the type and structure of the anionic moiety. In the literature, the effect of anions on biological activity was also considered. Generally, it is suggested that antimicrobial activity mainly depends on the length of the hydrocarbon chains of the compound, but some studies pointed out that the type of counterion also affects MIC [23,24]. Anion effects are of lesser importance than those of the cations. Not all literature data are consistent in this matter. It can be found that ILs containing Br^−^ have a stronger antimicrobial activity against *E. coli* than BF4^−^ and PF6^−^. The authors concluded that the hydrophobic anions BF4^−^ and PF6^−^ (unlike Br^−^) have poor solvation and dissociation. This is the reason for blocking the long alkyl chain of imidazolium from penetrating the bacterial membrane [19].

Moreover, and especially important in the context of our research, anions are involved in the interaction between water molecules and the phospholipid bilayer. Small hydrophilic anions cannot pass through the cell membrane (mainly because of the charge), while more hydrophobic anions are able to form a thin layer at the lipid–water interface. Furthermore, hydrophobic anions may be incorporated into the hydrocarbon tails of the membrane lipid bilayer to disorganize the phospholipid arrangements, with the cations located outside the membrane [19,25,26]. This effect is also likely in the case of the compounds with large anions tested by our team.

Contrary to our results, the anion showed almost no effect on antimicrobial activity, when taking into account imidazolium derivatives [8,27,28,29,30]. However, within the group of alkyltrihexylphosphonium ILs in the study [31], both cation structure and the type of anion had effects on the biological activity.

### 2.3. Surface Active Properties

**Figure 3:**
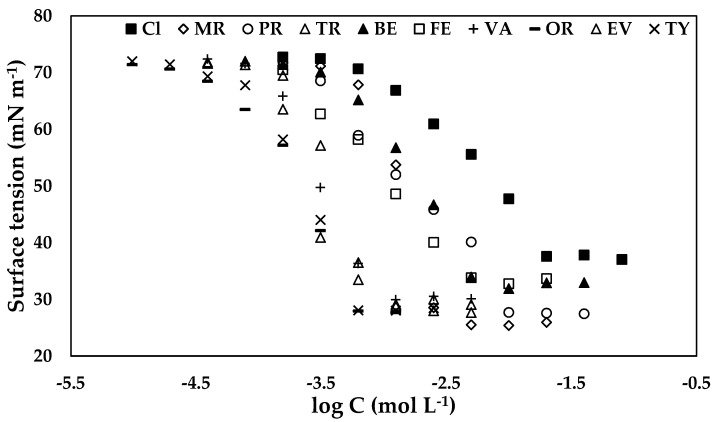
Surface tension vs. log C at 25 °C.

The surface activity of synthesized ionic liquids has been examined by surface tension measurements, which were obtained from the graphs in Figure 3. In general, the surface tension decreases with increasing IL concentration, until a plateau is reached. At this point, the surface tension value is constant. In fact, this is the common behavior of surface active ionic liquids that can adsorb at the liquid–air interface [32]. To investigate the surface activity of imidazolium ionic liquids, we used parameters such as critical micelle concentration (CMC), surface tension at the CMC (γCMC), maximum excess concentration (Γ_max_), free energy change of adsorption (ΔG^0^_ads_), and area per molecule residing at the surface (A_min_). The exact equations to determine the parameters Γ_max_, ΔG^0^_ads_, and A_min_ are described in a previous work [33]. Additionally, the wetting properties of the analyzed compounds were investigated. The results of the surface properties of ILs are summarized in Table 5.

The surface activity of ionic liquids can be evaluated by the effectiveness of lowering the surface tension of water (γCMC). Indeed, for aqueous solutions of ILs, the surface tension decreased from the value of water (72.8 mN m^−1^), to a minimum located from 25.2 to 37.7 mN m^−1^. This suggests that the molecules of imidazolium ionic liquids are able to aggregate in water solution [34]. Moreover, for the analyzed ILs, the micellization process was determined by the nature of the anion, although it is the cation that has an amphiphilic structure. In fact, the large anion can destabilize the micelle by steric hindrance. Basically, the surface activity depends on the structure of the ionic liquid, while, on the other hand it is related to the biostatic (bactericidal and fungicidal) activity. It has been confirmed in the literature that the ability of ILs to aggregate determines the rate of their diffusion to the cell surface and, thus, affects the permeability of the ionic liquid [35]. The CMC values (ranged from 0.72 to 32.35 mmol L^−1^) varied as expected, due to the change in hydrophobicity of the anion. It should be emphasized that the ability of ILs to inhibit microbial cell growth is not only determined by the structure of the ionic liquid, but also depends on biological factors, such as the above mentioned cell morphology or the physiological state of the microorganism.

While calculating surface parameters such as A_min_ (minimum surface area per molecule at the aqueous solution–air interface) and Γ_max_ (excess surface concentration), no clear analogy between these parameters was observed. Whereas the values obtained for ΔG^0^_ads_ were negative (for all ILs), which means that the adsorption phenomenon at the liquid–air interface is spontaneous.

Wettability was also studied, to determine the surface activity of imidazolium ionic liquids. Whereby, the CA values were in the range 0–90° (from 38.7°, which was equal for ILs with carvacrol acetate anion to 71.8° calculated for ILs with chloride anion). Based on the contact angle values obtained, it is concluded that the imidazolium salts can be classified as liquids that partially wet the hydrophobic paraffin surface.

### 2.4. Cytotoxic Properties

In our study, each of the 10 tested compounds exhibited cytotoxic properties against the mouse melanoma cell line (B16 F10) in the concentration range of the test (see Section 4). B16F10 is one of the most convenient cell models for both in vitro and in vivo experiments. This is due to the possibility of syngeneic tumor induction in an animal model, such implementation does not require sophisticated and expensive infrastructure (an IVC animal breeding facility). Thus, the choice of that cell line was due to the possibility of expanding research to an animal model in further studies. Table 6 presents average IC50 values, standard deviation (SD), and relative standard deviation (%RSD) for each of the tested compounds. Average IC50 values were in the range of 0.0101–0.0197 mM/L. Among the group of 10 compounds, TY-7 showed the strongest cytotoxic properties against the B16 F10 cell line. On the other hand, TR-10, MR-5, and CL-4 were less cytotoxic.

The comparison of cytotoxic and antimicrobial activity is ambiguous. In some cases cytotoxicity corresponded with antimicrobial activity, where TY-7 was the most active against both bacteria and mouse melanoma cells. The same relationship can be observed for compound EV-9. However, compounds CL-4 and PR-6, the most active against microbes, were the less cytotoxic compounds of all tested.

ILs can be used as potential anticancer agents, although knowledge about this remains limited. A general trend is found in the subject literature, that the toxicity of an IL can be related to the lipophilicity [6,18]. The cytotoxicity of ILs depends on their structure, and a major challenge is to build a structure with a high anticancer activity and low toxicity. Tests were carried out on vertebrates and invertebrates, including insects, fish, mice, and hamsters, as well as numerous human tumor cell lines, proving that derivatives of choline are less toxic than 1-alkyl-3-methylimidazolium and 1-alkylpyridinium [34,35,36]. Additionally, in the case of combinations of natural L-amino acids with the tetrafluoroborate anion the increase in cytotoxicity was most likely caused by the improved toxic transport of anions inside the cells. The antitumor activity and cytotoxicity of salts based on the phosphonium cation, ammonium, and 1-alkyl-3-methylimidazolium was tested on 60 lines of human tumor cells: leukemia, melanoma, and cancer of the lungs, colon, kidney, ovary, breast, prostate, and central nervous system; finding the relationship between the activity, toxicity, and length of the alkyl substituent. It has been shown that phosphonium derivatives are more active and less toxic than ammonium [37,38]. In the study of the action against human leukemia cell lines, the following compounds showed high activity: 1-dodecyl-3-methylimidazolium chloride and tetrafluoroborate; 1-hexadecyl-3-methylimidazolium chloride; and 1-octadecyl-3-methylimidazolium chloride, hexafluorophosphate, bis (trifluoromethylsulfonyl) imide, and tris (pentafluoroethyl)trifluorophosphate [39,40]. Some authors also analyzed the cytotoxic properties of benzalkonium chloride and didecyldimethylammonium chloride, which we used as standards for antimicrobial activity. It was found that the IC50 values for benzalkonium chloride were 0.19 mM/L for murine fibroblasts, 1.92 × 10^−2^ mM/L for lung epithelial cells, and 0.5–5 × 10^−3^ mM/L for Neuro2a cells and SK-N-SH cells, respectively [41,42]. On the other hand, IC50 values for didecyldimethylammonium chloride were 0.016 mM/L for A549 human alveolar epithelial adenocarcinoma cells and 1.57 × 10^−2^ mM/L for normal human lung cells [43].

The cytotoxicity of ILs v depends on the counterions used. However at present, there is no clear structure–cytotoxicity correlation, due to the different types of cell models used for research. It seems that Table 6 shows the anion does significantly not affect the cytotoxic properties in the conditions of our study. In [44], the bis(trifluoromethylsulfonyl)imide anion was clearly identified as a cytotoxicity-enhancing structural element, whereas the impact of the chloride, acetate, and tetrafluoroborate anions depended, both on the nature of the IL cation, and on the type of cell line being affected. The mechanisms of the cytotoxic action of ILs can vary and are not fully understood. However, taking into account their physicochemical properties it can be expected that, as in the case of antimicrobial activity, they damage the lipid–protein bilayer of the cell membrane, leading to necrosis of cancer cells [45,46]. Some authors have described other mechanisms, including alteration of cell membrane viscoelasticity; cell and nuclear membrane damage; mitochondrial permeabilization and dysfunction; generation of reactive oxygen species; alteration of transmembrane and cytoplasmatic protein/enzyme functions; alteration of signaling pathways; and DNA fragmentation [47].

### 2.5. Selectivity Indexes

An optimal structure of chemical compound requires a balance between antimicrobial properties and cell cytotoxicity. One of the issues concerning the development of many membrane-active agents as antibacterial agents is the selectivity for bacterial cells over mammalian cells, as these agents have the potential to disrupt human plasma membranes and induce related toxicity [48,49].

To compare the cytotoxic and antimicrobial activity, we calculated the selectivity indexes (SI) for each tested compound using data obtained for each microbial strain (Table 7).

The cytotoxicity is influenced by multiple physicochemical properties of the molecule, similarly to their MIC. All compounds exhibited SI values lower than 1; therefore, efforts will be required to improve the selectivity of these compounds.

## 3. Materials and Methods

All reagents, sodium hydroxide, chloroacetic acid, sulfuric acid, toluene, heptane, acetonitril, 1-methylimidazole, chloromethyloctyl ether, Dowex Monosphere 550A, formic acid, propionic acid, trimethylacetic acid, benzene sulfonic acid, 4-phenylsulfonic acid and phenol compounds (thymol, carvacrol, eugenol, vanilin) were obtained from Sigma-Aldrich, Poznań, Poland.

### 3.1. Synthesis

**Scheme 1:**
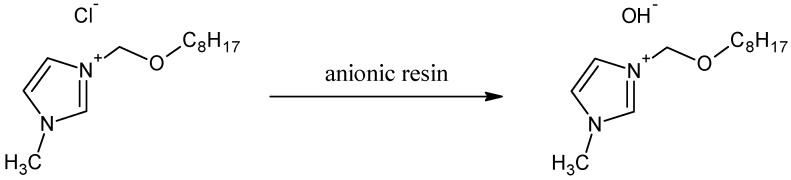
The synthesis of new ILs, alkalization process of 1-methyl-3-octyloxymethylimidazolium chloride.

**Scheme 2:**
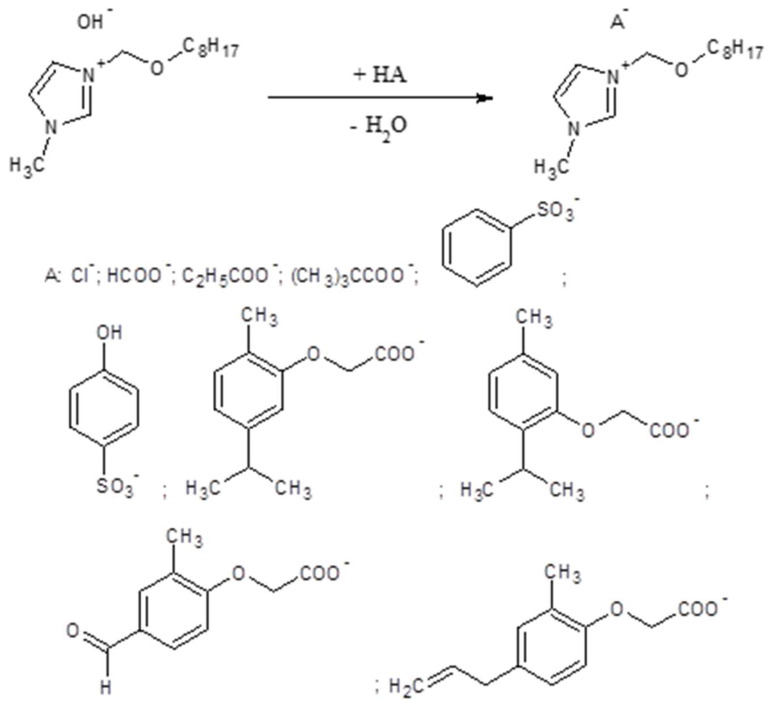
The synthesis of new ILs, neutralization process of 1-methyl-3-octyloxymethylimidazolium hydroxide.

**Scheme 3:**
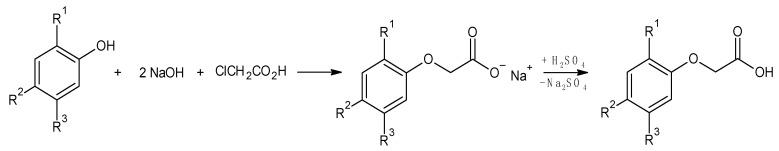
The synthesis of new ILs-phenoxy acid synthesis.

The new group of 10 imidazolium ionic liquids were prepared in three-step reactions, which can be found in Scheme 1, Scheme 2 and Scheme 3 and in Table 8.

In the first, 1-methyl-3-octyloxymethylimidazolium chloride was obtained in the Menschutkin reaction of 1-methylimidazole and chloromethyloctyl ether (1). The reaction was carried out for 1 h, in acetonitrile in 293 K, and the product was purified by extraction with heptane in 343 K (1). The product of 1-methyl-3-octyloxymethylimidazolium chloride was a hygroscopic compound with the yield 98.9%.

The second reaction was an alkalization process: 1-methyl-3-octyloxymethylimidazolium chloride was dissolved in water and passed through a column filled with a anionic resin Dowex Monosphere 550A.

The third reaction was a neutralization process of 1-methyl-3-octyloxymethylimidazolium hydroxide by proper acid HA (Scheme 2).

Next, water was removed using a rotary evaporator, and the obtained product was thoroughly dried under reduced pressure (5 mbar) at 60 °C for 10 h. All synthesized ILs were stored under reduced pressure over P4O10. The yield of neutralization reaction was 99.5%.

#### Phenoxy Acid Synthesis

A phenol compound (thymol, carvacrol, eugenol, vanilin) dissolved in warm water was added to a solution of sodium hydroxide (2.1 eq.) in warm water. After mixing for 30 min at 50 °C, monochloroacetic acid (2.1 eq.) was added. The obtained solution was mixed overnight at 50 °C. After cooling to room temperature, the solution was acidified to pH 2 with 10% sulfuric acid. The obtained solid phenoxy acetic acid was separated with a Buchner funnel. Water from the aqueous phase was removed on a rotary evaporator, and the residue was dissolved in hot toluene and washed with water. After separation from the organic phase, phenoxyacetic acid was precipitated and separated with a Buchner funnel. The combined crude phenoxyacids were dissolved in hot toluene, and activated charcoal was added and mixed for 15 min at 50 °C. After removing the charcoal from the hot solution, the organic phase was cooled to room temperature, and the obtained solid phenoxyacetic acid was filtrated and dried in vacuum.

### 3.2. Characterization of New ILs

#### 3.2.1. NMR and IR Spectroscopy

The novel group of imidazolium based ionic liquids was characterized by ^1^H and ^13^C-NMR spectroscopy. ^1^H and ^13^C NMR spectra were recorded at 25 °C using a 400 MHz (for ^1^H) and 100 MHz (for ^13^C) Bruker NMR spectrometer. The infrared (IR) spectra were obtained on a Nexus Nicolet 5700 Fourier Transform Infrared Spectrophotometer (FTIR, Thermo Electron Scientific Instruments Corporation, Madison, WI, USA) equipped with an attenuated total reflection (ATR) accessory, with a diamond crystal (T = 25 °C, range 4000–600 cm^−1^, resolution 4 cm^−1^ at 64 scans). Results can be found in the Supplementary Section File.

#### 3.2.2. Thermal Properties

Thermal transition temperatures were determined by DSC, with a Mettler Toledo Stare DSC1 (Leicester, UK) unit, under nitrogen. Samples (between 5 and 15 mg) were placed in aluminum pans and heated from 25 to 110 °C, at a heating rate of 10 °C/min, cooled with an intracooler at a cooling rate of 10 °C/min to −100 °C, then heated again to 120 °C. Thermogravimetric analysis was performed using a Mettler Toledo Stare TGA/DSC1 unit (Leicester, UK), under nitrogen. ILs (between 2 and 10 mg) were placed in aluminum pans and heated from 30 to 450 °C at a heating rate of 10 °C/min. The thermal analysis apparatus was 605 calibrated by measuring the following standards: In (purity 606 99.999%, Tm = 156.49 °C, ΔH = 29.38 J/g), Pb (purity 99.99%, Tm = 327 °C, ΔH =22.25 J/g), Zn (purity 99.998%, Tm = 418.78 °C, ΔH = 106.53 J/g), Al (purity 99.99%, Tm = 660.03 °C, ΔH = 340.15 J/g).

### 3.3. Surface Active Properties Determination

Surface tension and contact angle measurements were carried out with the use of a DSA 100 analyzer (Krüss, Germany, the accuracy of 0.01 mN m^−1^) at 25 °C. Temperature was controlled using a Fisherbrand FBH604 thermostatic bath (Fisher, Germany, accuracy 0.1 °C). The surface tension was determined using the shape drop method. Generally, this method is based on the analysis of an asymmetric drop situated at the tip of a syringe needle. During the whole measurement, an image of the drop (3 mL) is taken from a CCD camera and digitized. The surface tension (γCMC) is calculated by analyzing the profile of the drop, according to the mathematic Laplace equation. The values of parameters such as critical micelle concentration (CMC) and the surface tension at the CMC (γCMC) were determined from the intersection of two straight lines drawn in the low and high concentration regions in surface tension curves (γCMC vs. log C curves) using a linear regression analysis method.

The contact angle (CA) was determined using the sessile drop method. The concentration of liquid drops that were deposited on the solid (paraffin) surface was above CMC. In this work, we used paraffin, because it is a highly hydrophobic surface. After identification of actual drop shape and correct contact line, the drop was fit to the Young–Laplace equation. The CA was determined as the slope of the contour line at the 3-phase contact point (solid–liquid and liquid–air).

### 3.4. Antimicrobial Activity Determination

The antimicrobial activity of the synthesized compounds was tested against: *Staphylococcus aureus* ATCC 25213, *Pseudomonas aeruginosa* ATCC 27853, *Klebsiella pneumoniae* ATCC 700603, *Escherichia coli* ATCC 25922, *Enterococcus faecalis* ATCC 29212, and *Candida albicans* ATCC 90028. The strains were purchased from Microbiologics (San Diego, CA, USA). Bacterial, fungal suspensions with a turbidity equivalent to that of a 0.5 McFarland standard were prepared by suspending growth from blood Trypticase Soy Agar (TSA, BBL) plates in 2 mL of sterile saline. The antibacterial effects of the analyzed compounds were evaluated by determination of minimal inhibitory concentration (MIC, % *w*/*v*) values using the disk-diffusion method, ranging from 1.0 to 0.000125 and the control plate without disinfectant. The test medium was Mueller–Hinton agar (bio-Merieux). The MIC was determined as the lowest concentration of the tested agent at which no visible bacterial growth could be detected. A barely visible haze of growth was disregarded. MIC was read at 37 °C, after 24 or 48 h culture for the other bacteria and for Candida spp. Details of the agar dilution procedure for testing microorganisms have been published by the Clinical and Laboratory Standards Institute [15].

### 3.5. Cytotoxic Activity Determination

#### 3.5.1. Cell Culture of the B16 F10 Line

Mouse melanoma cell line (B16 F10) was kindly provided by Prof. Tomasz Drewa, a Chair of Urology, Department of Regenerative Medicine, Collegium Medicum, Nicolaus Copernicus University, Bydgoszcz, Poland. All cell culture procedures were performed under aseptic conditions in a biosafety cabinet. Cells were cultivated in 2–3 splitting cycles per week and were maintained at 37 °C in a 5% CO_2_ atmosphere with 95% humidity. The B16 F10 cell line was cultured in RPMI cell culture medium (with l-glutamine and sodium bicarbonate, Sigma-Aldrich) supplemented with 10% of fetal bovine serum (FBS, Biowest, Nuaillé, France), as well as antibiotics and antimycotics (Sigma-Aldrich).

#### 3.5.2. Determination of Cytotoxicity Using the MTT Test

The number of cells was determined automatically with a Countess™ II FL Automated Cell Counter (Invitrogen by Thermo Fisher Scientific Inc., Waltham, MA, USA). Cells were seeded in a 96-well plate (Nunc Edge 2F, Thermo-Fisher Scientific, Waltham, MA, USA) at an inoculum of 1000 cells per well. After 24 h, test compounds were added, with final concentrations as described in the ‘cytotoxicity assessment’ paragraph. After 72 h of incubating cells with test compounds, 20 µL of MTT solution (5 mg/mL) was added to each well. After 2 h, the medium was filtered off, and the obtained formazan crystals were dissolved in 100 μL of pure isopropanol. Immediately, absorbance readout was performed at 570 nm and 690 nm with a UV-VIS plate spectrophotometer (Multiskan Spektrum, Thermo Electron Corporation, Waltham, MA, USA). If not stated otherwise all chemicals were purchased from Sigma-Aldrich.

#### 3.5.3. Cytotoxicity Assessment

The effect of 10 compounds on the survival rate of the cell line B16 F10 was determined. Each sample was dissolved with water, to obtain stock solutions of 1 mg/mL. Further dilutions were made with the cell culture medium. The test was carried out at the following final concentrations: 10 µg/mL; 1 µg/mL; 0.1 µg/mL; and 0.01 µg/mL. The test solutions were applied to cells in tetraplicates. Using the ED50plus v 1.0 freeware software (Instituto Nacional de Enfermedades Respiratorias, Mexico D.F., Mexico), the IC50 parameter was determined. The experiment was carried out in three independent biological replicates. In this study we did not analyze any standards, as our aim was to demonstrate the cytotoxicity of the pilot set of newly proposed compounds, and to estimate their overall potential and to indicate future directions of study.

### 3.6. Determination of the Selectivity Index (SI)

Selectivity indexes (SI) were calculated for the analyzed compounds, taking into account the MIC against *Staphylococcus aureus* ATCC 25213, *Pseudomonas aeruginosa* ATCC 27853, *Klebsiella pneumoniae* ATCC 700603, *Escherichia coli* ATCC 25922, *Enterococcus faecalis* ATCC 29212, *Candida albicans* ATCC 90028, and the IC50 on the mouse melanoma cell line (B16 F10) (SI=IC50/MIC).

## 4. Conclusions

In this paper we described an effective method of obtaining 10 new ionic liquids with 1-methyl-3-octyloxymethylimidazolium cation and various anionic moieties. Synthesis of all compounds was performed in high yield. This group of compounds shows antimicrobial and cytotoxic effects. The lowest MIC values were obtained for *Candida albicans* and *Staphylococcus aureus* strains; whereas, *Pseudomonas aeruginosa* was the most resistant strain. Moreover, it was found that TY-7 had the highest cytotoxic activity. There were no clear dependencies between antimicrobial and cytotoxic activity, and surface properties. At this stage, it could be concluded that the most potent compounds, containing thymoloxyacetate and eugenoloxyacetate moiety, could be candidates for new antimicrobial agents or surfactants.

## Figures and Tables

**Table 1:** Chemical Structure of Anions of New ILs.

Name	Anion Structure
**FE-1**	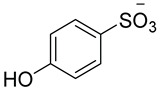
**BE-2**	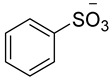
**OR-3**	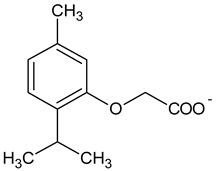
**CL-4**	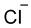
**MR-5**	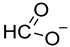
**PR-6**	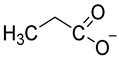
**TY-7**	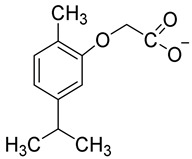
**VA-8**	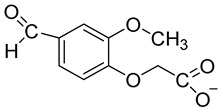
**EV-9**	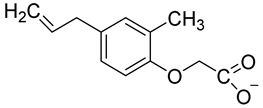
**TR-10**	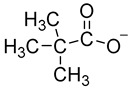

**Table 2:** DSC data for obtained ionic liquids.

Compound	Tg (°C)	Tc (°C)	Tm (°C)
**FE-1**	−49.76	−23.61	8.8
**BE-2**	−29.37	−4.78 −0.56	−18.90 17.10
**OR-3**	−69.24	−50.39	−43.05
**CL-4**	-	−2.02	16.62
**MR-5**	−54.54	−18.75	−24.98
**PR-6**	-	−52.23	−37.01
**TY-7**	−68.73	-	-
**VA-8**	−36.92	-	-
**EV-9**	−59.45	−49.36	−37.26
**TR-10**	-	−68.65	−61.58

Tm—melting point; Tc—temperature of crystallization; Tg—glass transition temperature.

**Table 3:** Thermal stability for obtained ionic liquids.

Compound	Ti (°C)	T0.01 (°C)	T0.02 (°C)	T0.05 (°C)
**FE-1**	138.65	159.92	170.90	192.18
**BE-2**	135.62	163.71	176.93	196.93
**OR-3**	115.98	130.23	139.40	156.37
**CL-4**	117.60	147.81	159.69	177.68
**MR-5**	97.48	107.77	116.00	129.73
**PR-6**	85.81	97.92	106.05	120.46
**TY-7**	114.24	133.92	144.79	162.77
**VA-8**	112.85	135.25	144.08	157.66
**EV-9**	118.62	135.59	145.78	163.09
**TR-10**	102.67	114.21	122.36	134.91

Ti, initial decomposition temperature at which dm/dt obtained value 10–4 mg/s; T0.01, decomposition temperature of 1% sample; T0.02, decomposition temperature of 50% sample; T0.05, decomposition temperature of 50% sample

**Table 4:** MIC values of the group of new ILs.

Compound	MIC (mM/L)					
	SAU	KPN	PAE	ECO	EFA	CAL
**FE-1**	1.7248	13.9097	27.8195	1.7248	1.7248	1.7248
**BE-2**	0.2329	1.8052	7.2788	0.4658	0.2329	0.2329
**OR-3**	0.1016	1.5754	6.3526	1.5754	0.1016	0.1016
**CL-4**	0.1804	1.3980	5.6371	0.3608	0.1804	0.1804
**MR-5**	0.1729	2.6804	10.8079	0.3459	0.1729	0.1729
**PR-6**	0.1542	1.1952	4.8195	0.1542	0.3084	0.1542
**TY-7**	0.0508	0.7877	3.1763	0.4066	0.0508	0.1016
**VA-8**	0.1011	1.5678	6.3216	0.4046	0.1011	0.2023
**EV-9**	0.0982	3.0673	3.0673	1.5214	0.0982	0.1963
**TR-10**	0.2783	2.1571	8.6981	0.5567	0.2783	0.2783
**ST-1**	0.0035	0.8561	0.8561	0.0138	0.0069	0.0069
**ST-2**	0.0284	0.4555	1.7653	0.0569	0.0142	0.0142

**Table 5:** Surface active parameters of imidazolium ILs.

No	CMC [mmol L^−1^]	γ_CMC_ (mN m^−1^)	Γ_max_ (mol m^−2^)	A_min_ × 10^−19^ (m^2^)	ΔG^0^_ads_ × 10^4^ (J m^−1^)	Contact Angle (°)
**FE-1**	4.78	33.5	7.84 × 10^6^	2.12	−1.84	61.2
**BE-2**	6.11	32.0	1.33 × 10^5^	1.25	−1.50	58.9
**OR-3**	0.87	27.9	7.69 × 10^6^	2.16	−2.26	38.7
**CL-4**	32.35	37.7	6.92 × 10^6^	2.40	−1.43	71.8
**MR-5**	2.99	25.2	1.63 × 10^5^	1.02	−1.53	62.5
**PR-6**	15.63	27.6	5.53 × 10^6^	3.00	−1.89	64.7
**TY-7**	0.82	28.2	8.72 × 10^6^	1.91	−2.01	39.5
**VA-8**	0.82	30.1	1.19 × 10^5^	1.39	−1.96	53.8
**EV-9**	0.96	29.5	1.27 × 10^5^	1.31	−1.93	48.9
**TR-10**	0.72	28.8	8.78 × 10^6^	1.89	−2.15	46.8

**Table 6:** Cytotoxic properties of the analyzed compounds.

	FE-1	BE-2	OR-3	CL-4	MR-5	PR-6	TY-7	VA-8	EV-9	TR-10
IC 50 [mM/L]	1.22 × 10^−2^	1.22 × 10^−2^	1.11 × 10^−2^	1.97 × 10^−^^2^	1.84 × 10^−2^	1.58 × 10^−2^	1.01 × 10^−2^	1.11 × 10^−2^	1.11 × 10^−2^	1.63 × 10^−2^
SD	5.02 × 10^−4^	9.42 × 10^−4^	9.47 × 10^−4^	3.80 × 10^−3^	8.49 × 10^−4^	2.55 × 10^−3^	5.62 × 10^−4^	1.05 × 10^−3^	8.34 × 10^−4^	5.09 × 10^−4^
% RSD	4.11	7.74	8.51	19.29	4.62	16.09	5.56	9.42	7.55	3.13

**Table 7:** Selectivity indexes.

Compound	SI_SAU_	SI_KPN_	SI_PAE_	SI_ECO_	SI_EFA_	SI_CAL_
**FE-1**	0.0071	0.0009	0.0004	0.0071	0.0071	0.0071
**BE-2**	0.0524	0.0068	0.0017	0.0262	0.0524	0.0524
**OR-3**	0.1096	0.0071	0.0018	0.0071	0.1096	0.1016
**CL-4**	0.1092	0.0141	0.0035	0.0546	0.1092	0.1092
**MR-5**	0.1064	0.0069	0.0017	0.0532	0.1064	0.1064
**PR-6**	0.1025	0.0132	0.0033	0.1025	0.0512	0.1025
**TY-7**	0.1988	0.0128	0.0032	0.0248	0.1988	0.0994
**VA-8**	0.1098	0.0071	0.0018	0.0274	0.1098	0.0549
**EV-9**	0.1130	0.0036	0.0036	0.0073	0.1130	0.0565
**TR-10**	0.0586	0.0076	0.0019	0.0293	0.0586	0.0586

**Table 8:** Details of phenoxy acid synthesis.

	R^1^	R^2^	R^3^	Yield (%)	Mp. (°C)
Thymoloxyacetic acid	iPr	H	Me	87	146
Carvacroloxyacetic acid	Me	H	iPr	82	151
Vanillinoxyacetic acid	OMe	CHO	H	72	190 *
Eugenoloxyacetic acid	OMe	All	H	76	99

* Recrystallized from 96% Ethanol.

## Data Availability

The article contains data supporting the findings.

## Supplementary Materials

The following supporting information can be downloaded at: https://www.mdpi.com/article/10.3390/molecules27061974/s1, Section S1.1: FTIR ATR spectra; Section S1.2: DSC data; Section S1.3: Thermal stability and TGA curves; Section S1.4: Elementary analysis; Section S1.5: ^1^H and ^13^C-NMR spectroscopy.

## Sample Availability

Samples of the compoundsare not available from the authors.
