# Supplementary material for: Antimicrobial and Cytotoxic Activity of Novel Imidazolium-Based Ionic Liquids
Source: Molecules. 2022 Mar 18;27(6):1974. doi: 10.3390/molecules27061974 (PMC8953206; doi:10.3390/molecules27061974)
Supplement: Supplementary file 1 [file molecules-27-01974-s001.zip › molecules-1532617-supplementary.pdf]

Antimicrobial and cytotoxic activity of novel imidazolium-based ionic liquids

Łukasz Pałkowski, Maciej Karolak, Andrzej Skrzypczak, Marta Wojcieszak, Filip Walkiewicz, Jonasz Podemski, Karol Jaroch, Barbara Bojko, Katarzyna Materna, Jerzy Krysiński

Supplementary material

Section S1.1. FTIR ATR spectra

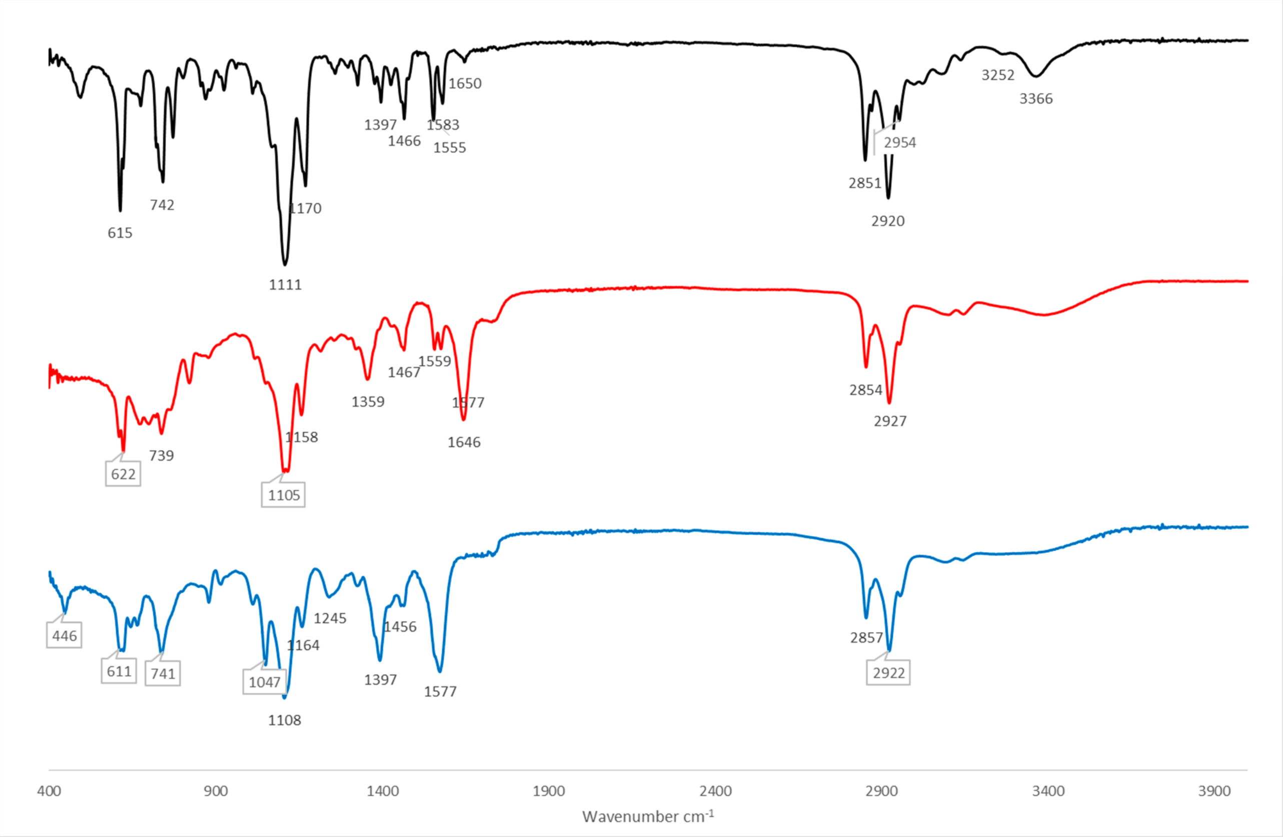

Figure S1. FTIR ATR spectra for CL-4 (black) MR-5 (red) and PR-6 (blue),

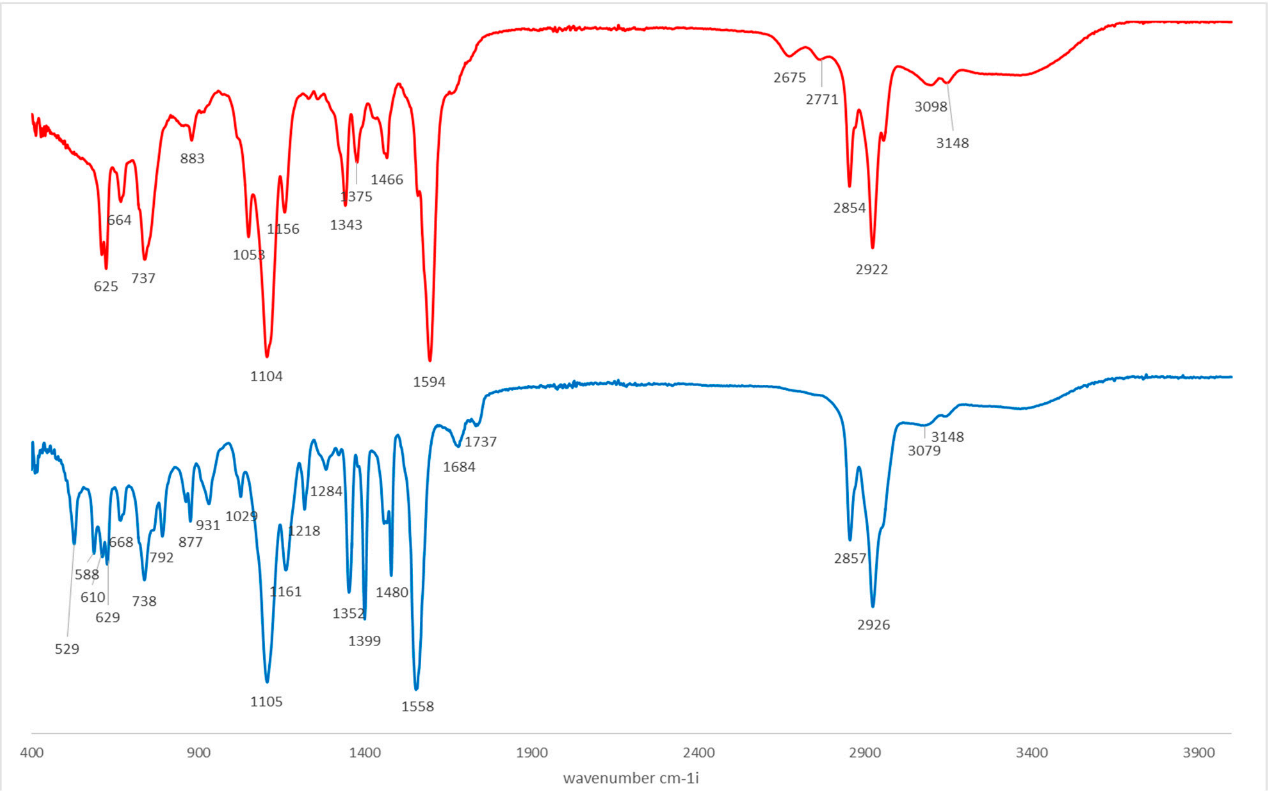

Figure S2. FTIR ATR spectra for TR-10 (red) compared with dichlotoacetate (blue),

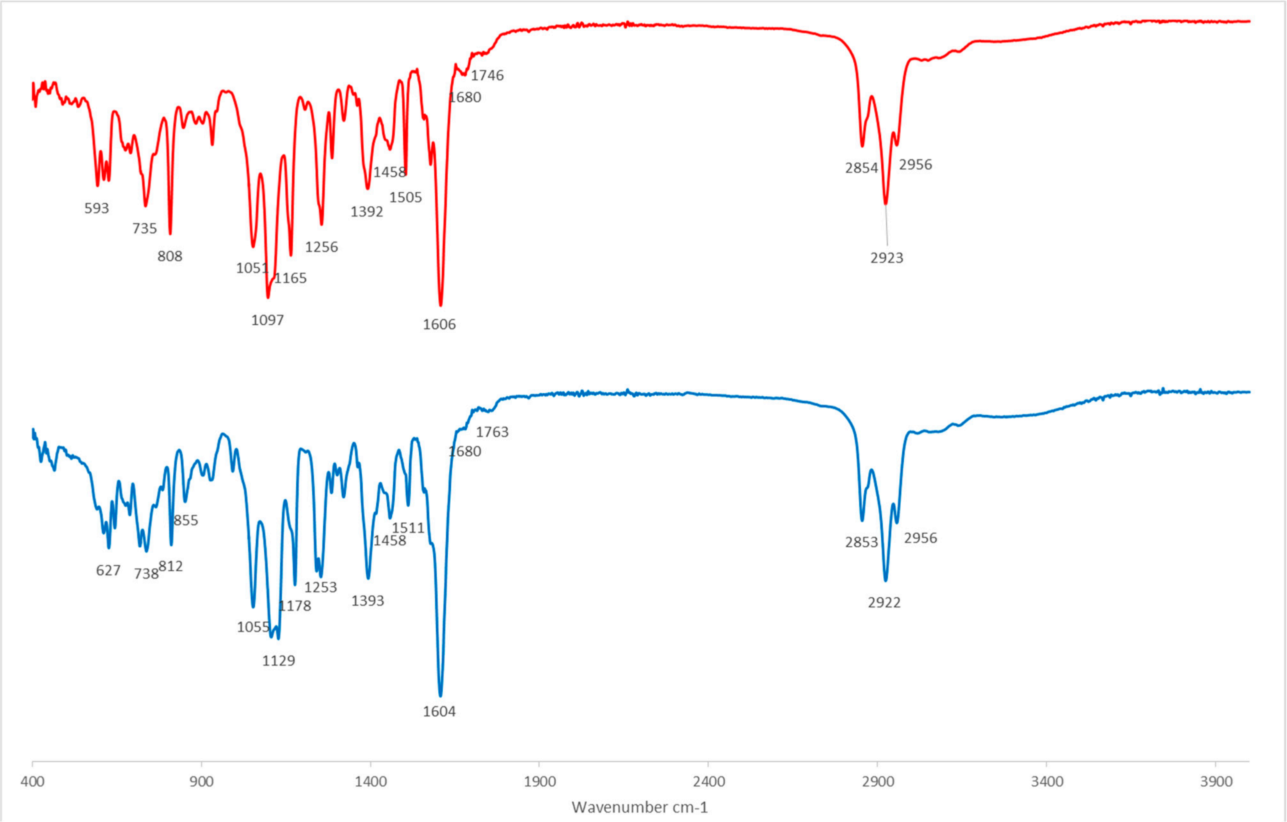

Figure S3. FTIR ATR spectra for TY-7 (red) and OR-3 (blue),

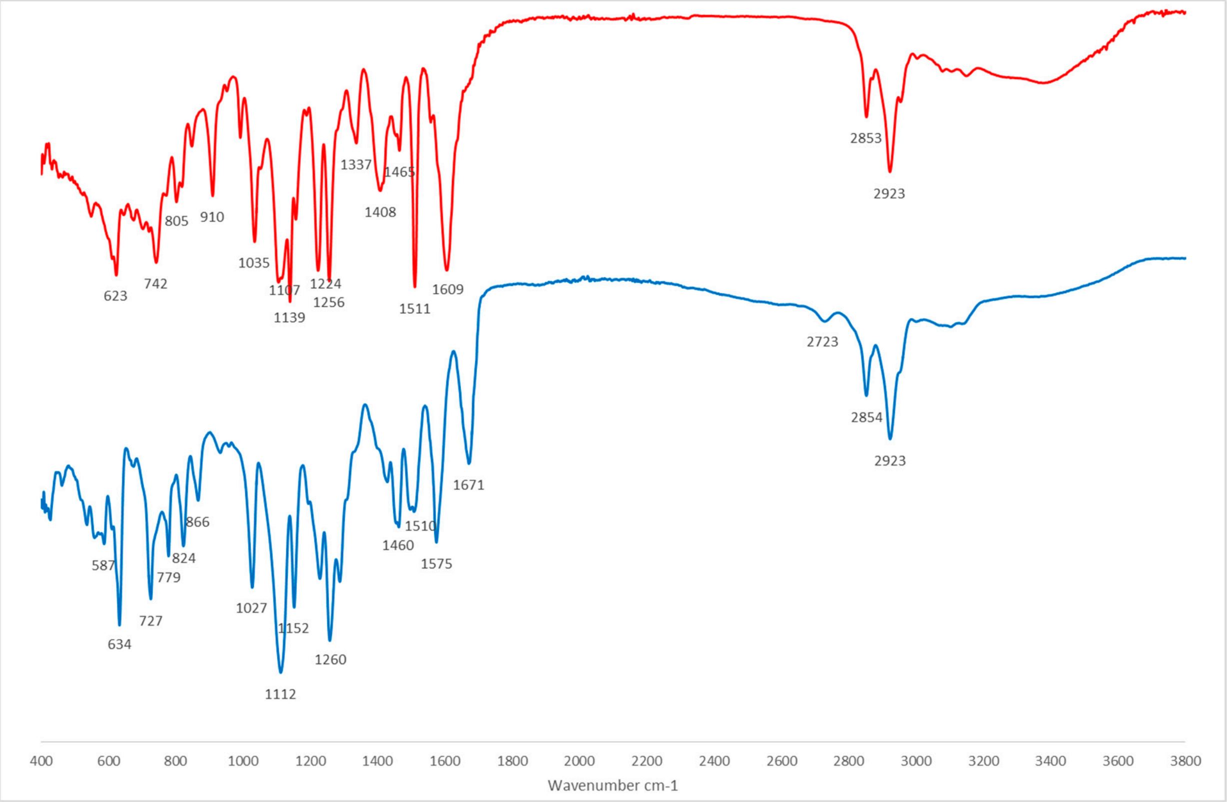

Figure S4. FTIR ATR spectra for EV-9 (red) and VA-8 (blue),

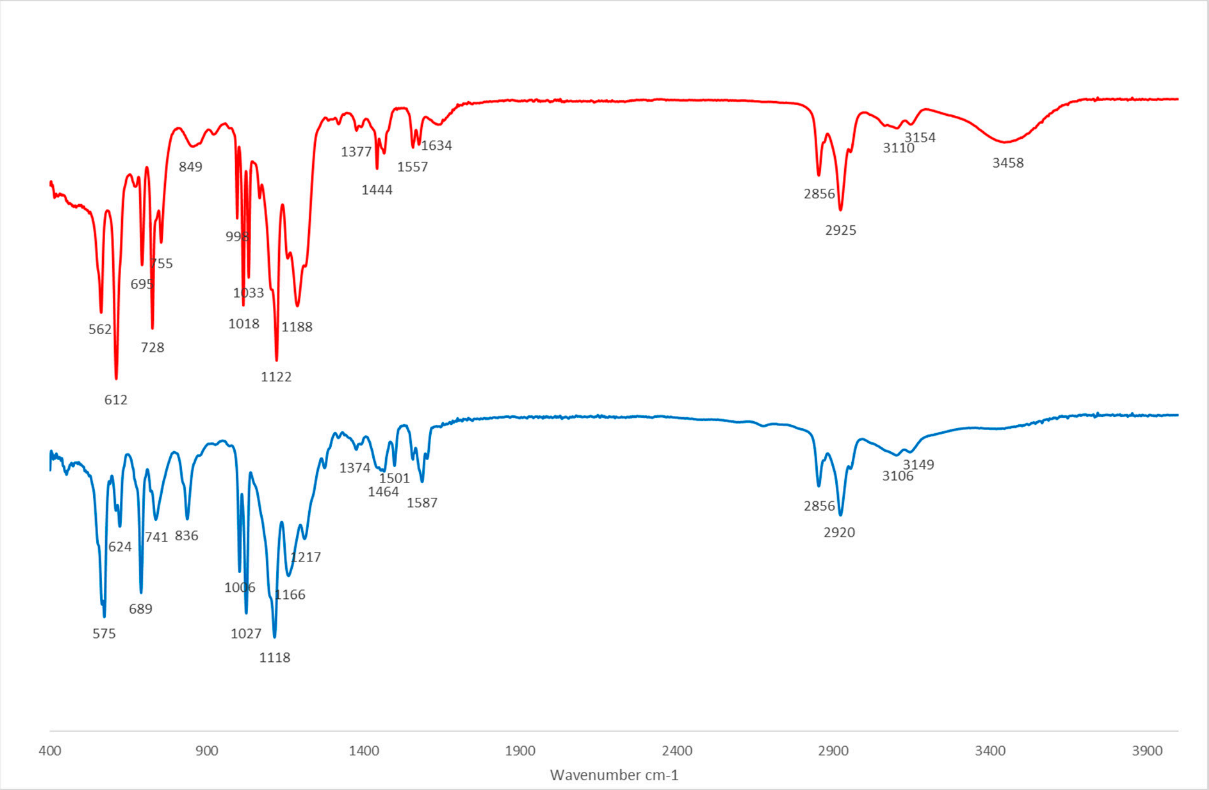

Figure S5. FTIR ATR spectra for FE-1 (red) and BE-2 (blue),

Section S1.2. DSC data

Table S1. DSC data for obtained ionic liquids

compound	T_g [°C][a]	T_c [°C][b]	T_m [°C][c]
FE-1	-49.76	-23.61	8.8
BE-2	-29.37	-4.78<br>-0.56	-18.90<br>17.10
OR-3	-69.24	-50.39	-43.05
CL-4	-	-2.02	16.62
MR-5	-54.54	-18.75	-24.98
PR-6	-	-52.23	-37.01
TY-7	-68.73	-	-
VA-8	-36.92	-	-
EV-9	-59.45	-49.36	-37.26
TR-10	-	-68.65	-61.58

Tm – melting point; Tc – temperature of crystallization; Tg – glass transition temperature

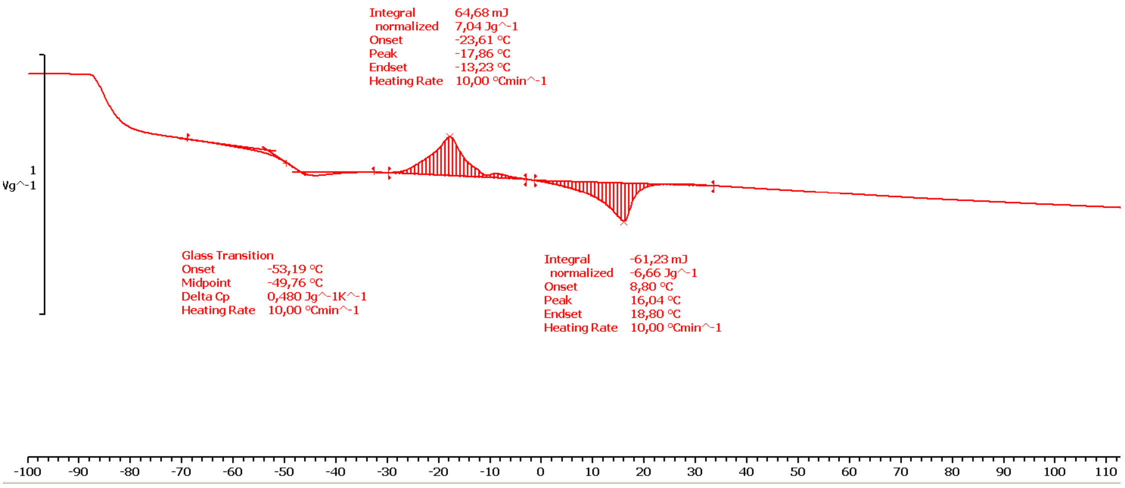

DSC curve for FE-1

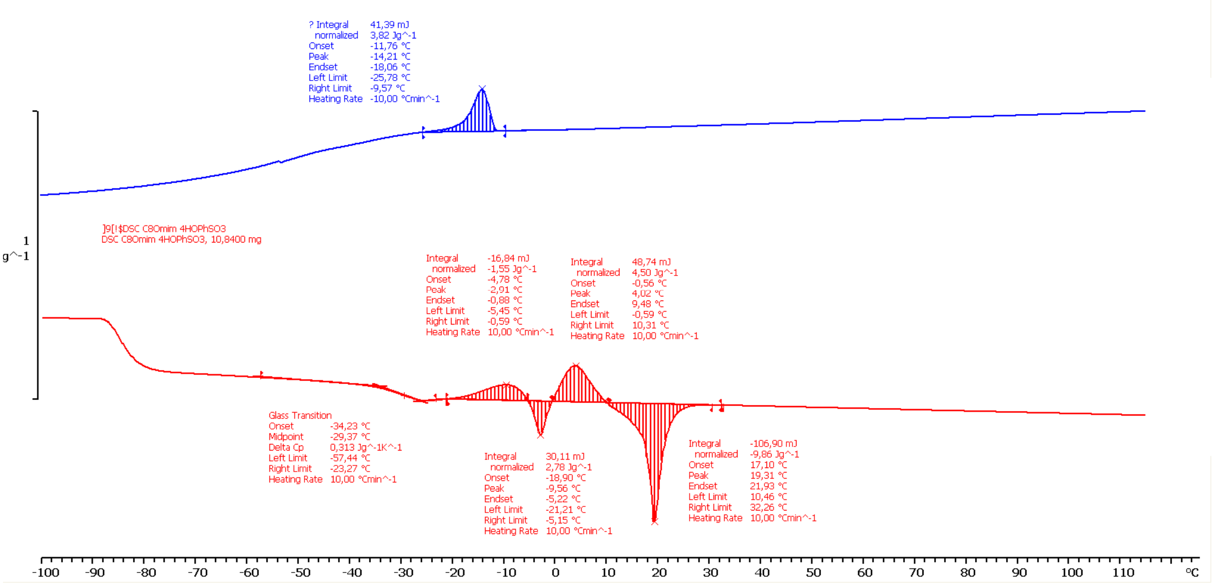

DSC curve for BE-2

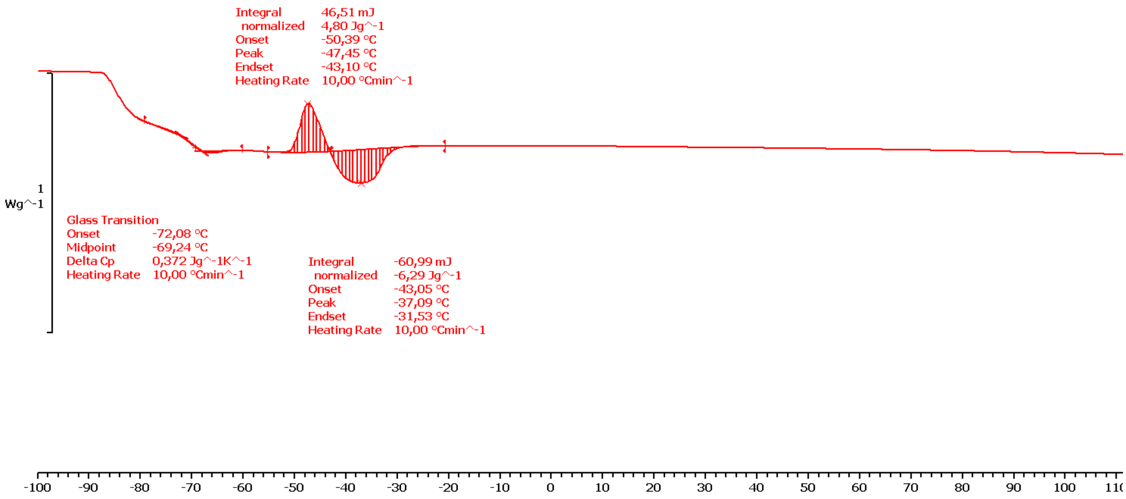

DSC curve for OR-3

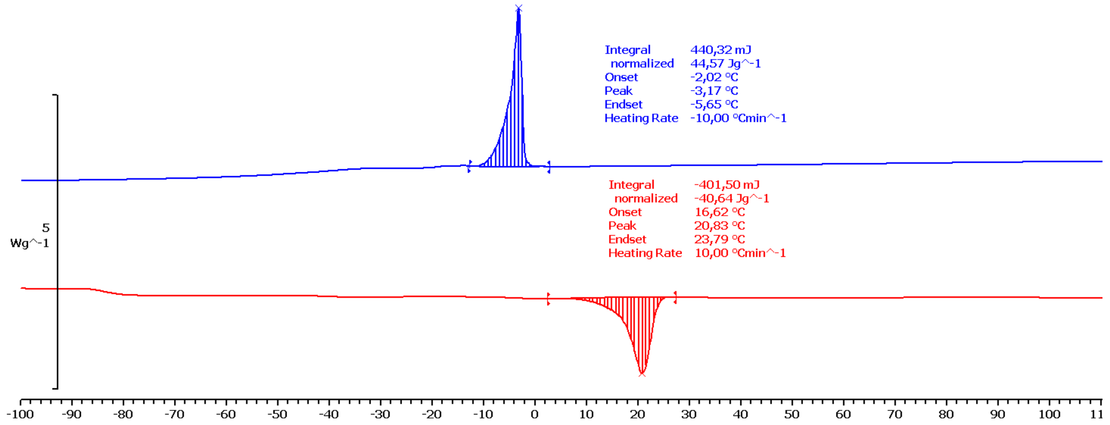

DSC curve for CL-4

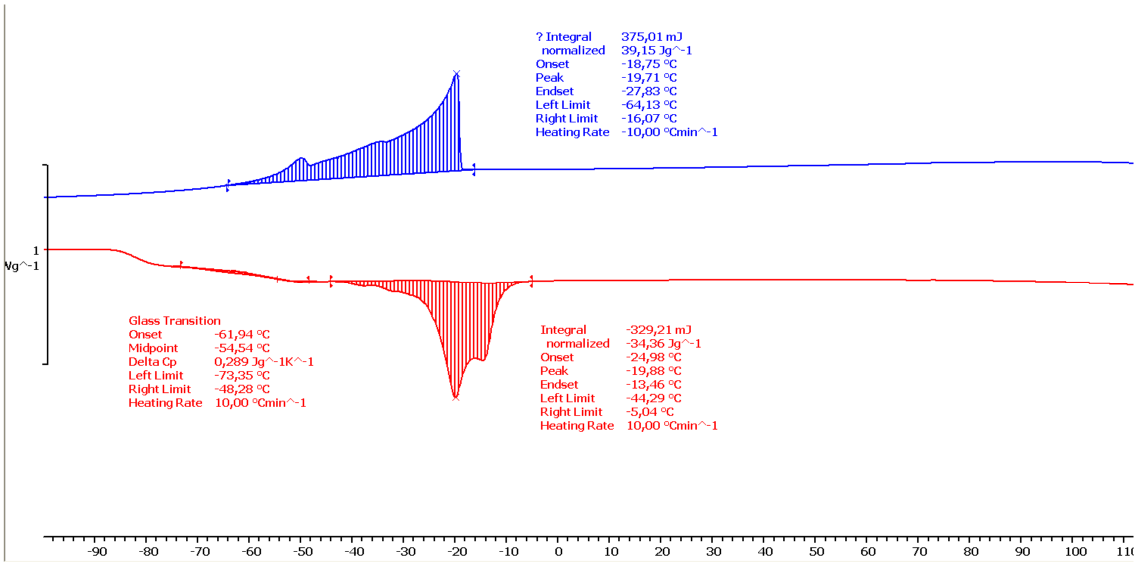

DSC curve for MR-5

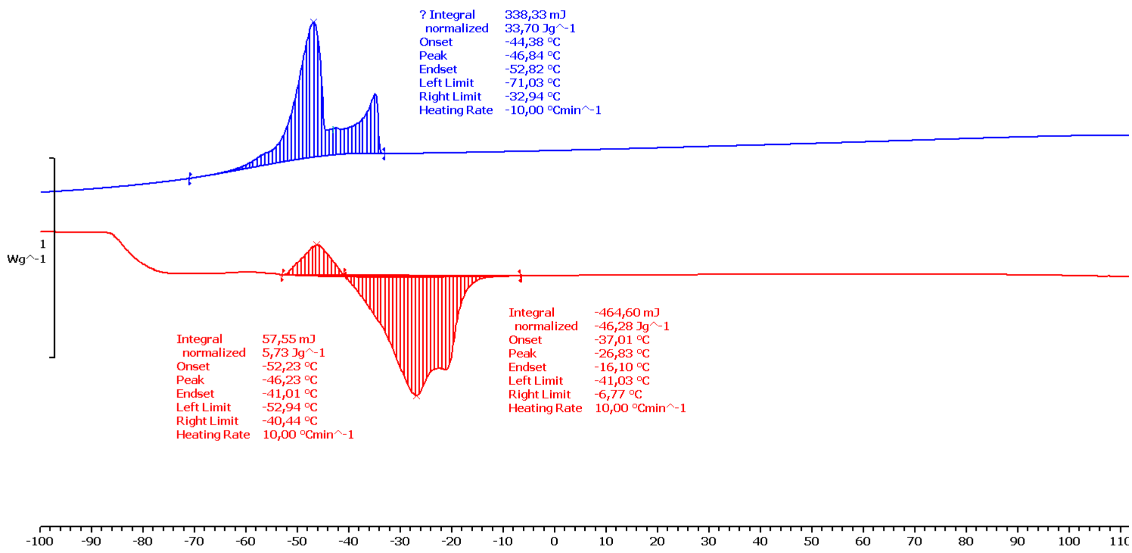

DSC curve for PR-6

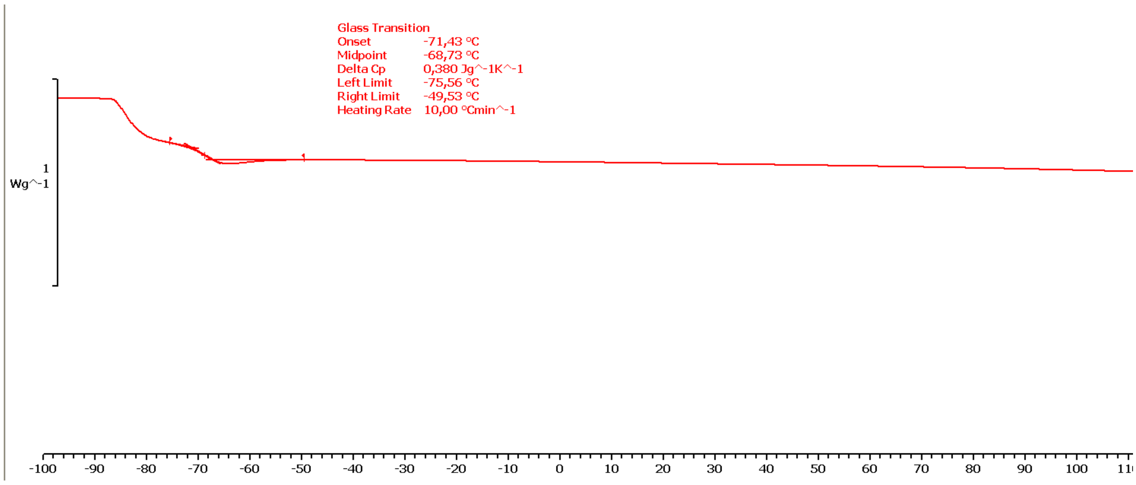

DSC curve for TY-7

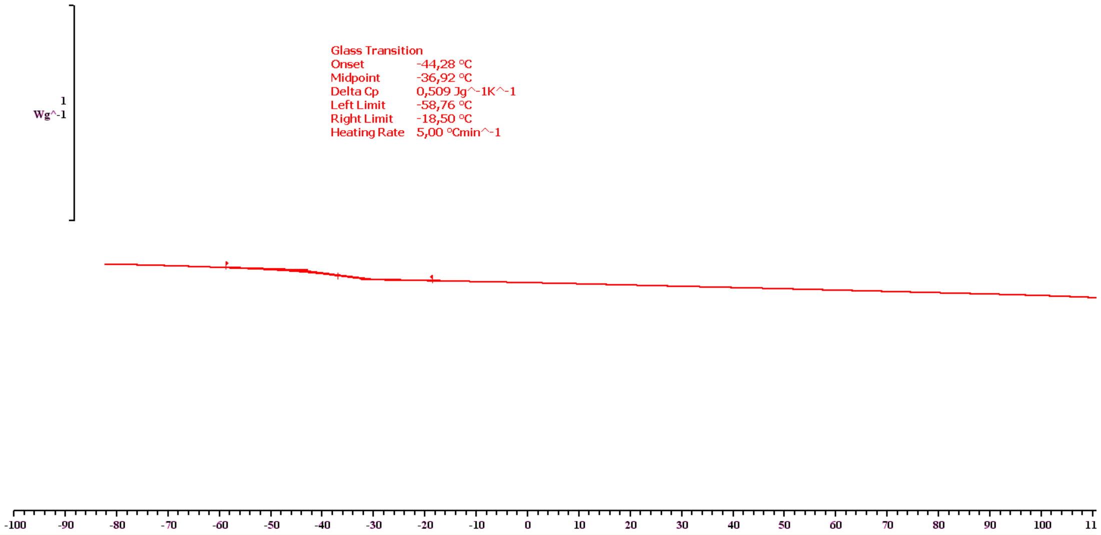

DSC curve for VA-8

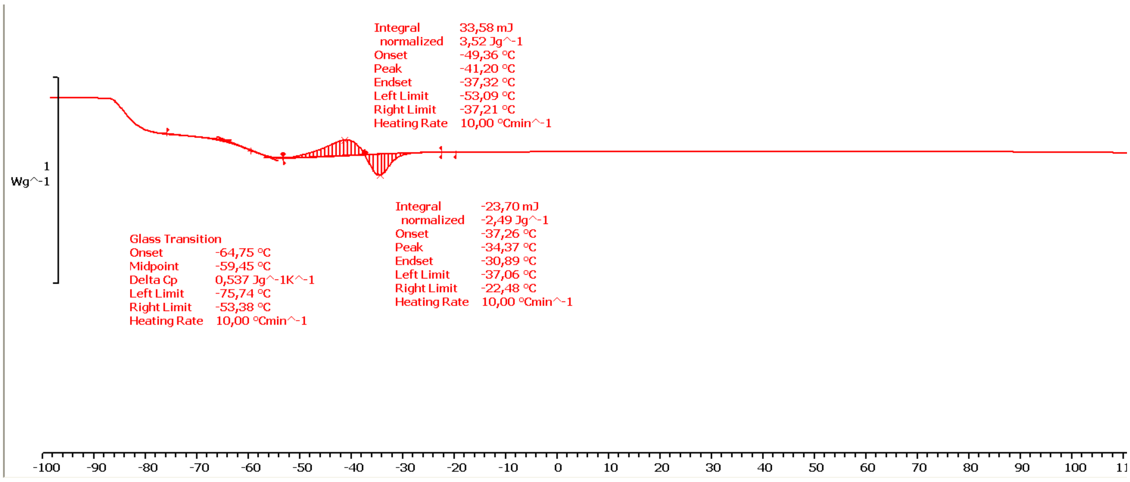

DSC curve for EV-9

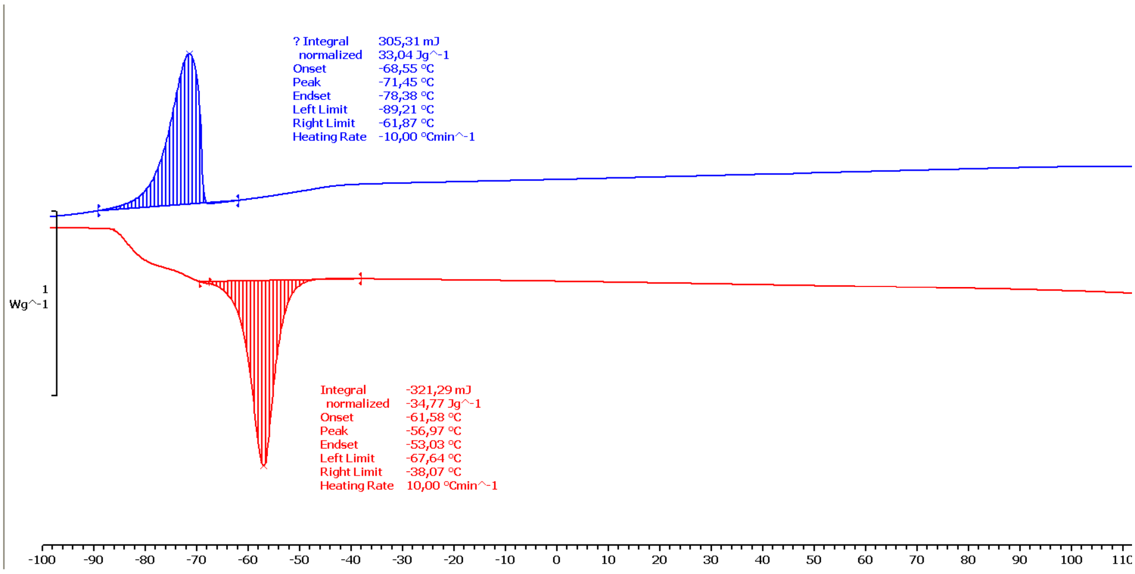

DSC curve for TR-10

Section S1.3. Thermal stability and TGA curves

Table S2. Thermal stability for obtained ionic liquids

compound	T_i [°C]	$T_{0.01}$ [°C]	$T_{0.02}$ [°C]	$T_{0.05}$ [°C]
FE-1	138.65	159.92	170.90	192.18
BE-2	135.62	163.71	176.93	196.93
OR-3	115.98	130.23	139.40	156.37
CL-4	117.60	147.81	159.69	177.68
MR-5	97.48	107.77	116.00	129.73
PR-6	85.81	97.92	106.05	120.46
TY-7	114.24	133.92	144.79	162.77
VA-8	112.85	135.25	144.08	157.66
EV-9	118.62	135.59	145.78	163.09
TR-10	102.67	114.21	122.36	134.91

T_i initial decomposition -temperature at which dm/dt obtained value 10^{-4} mg/s

$T_{0.01}$ – decomposition temperature of 1% sample;

$T_{0.02}$ – decomposition temperature of 50% sample

$T_{0.05}$ – decomposition temperature of 50% sample

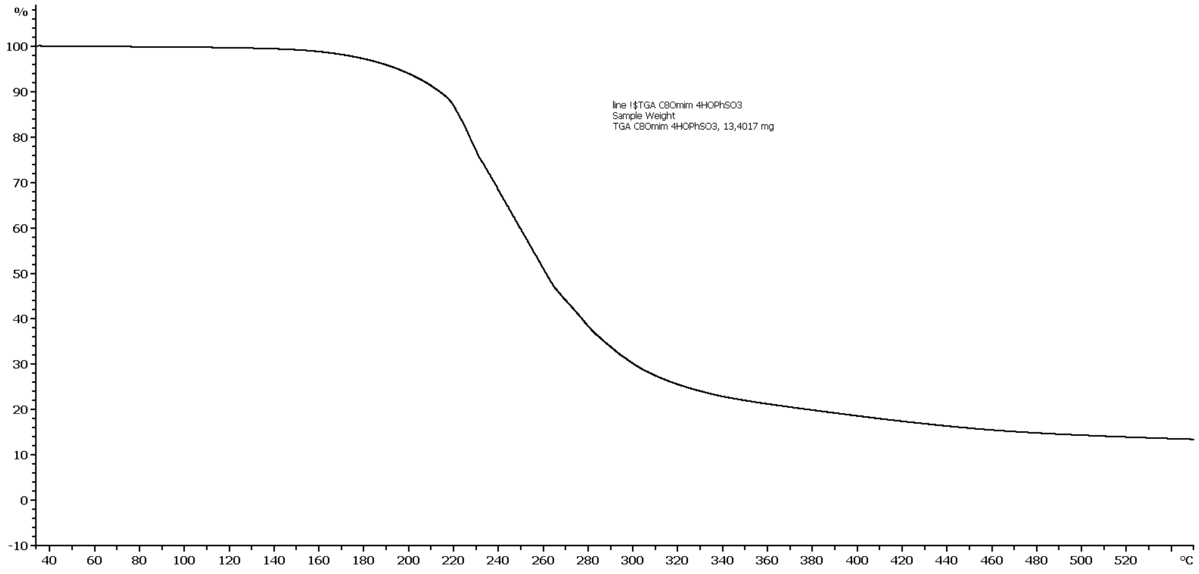

TGA curve for compound FE-1

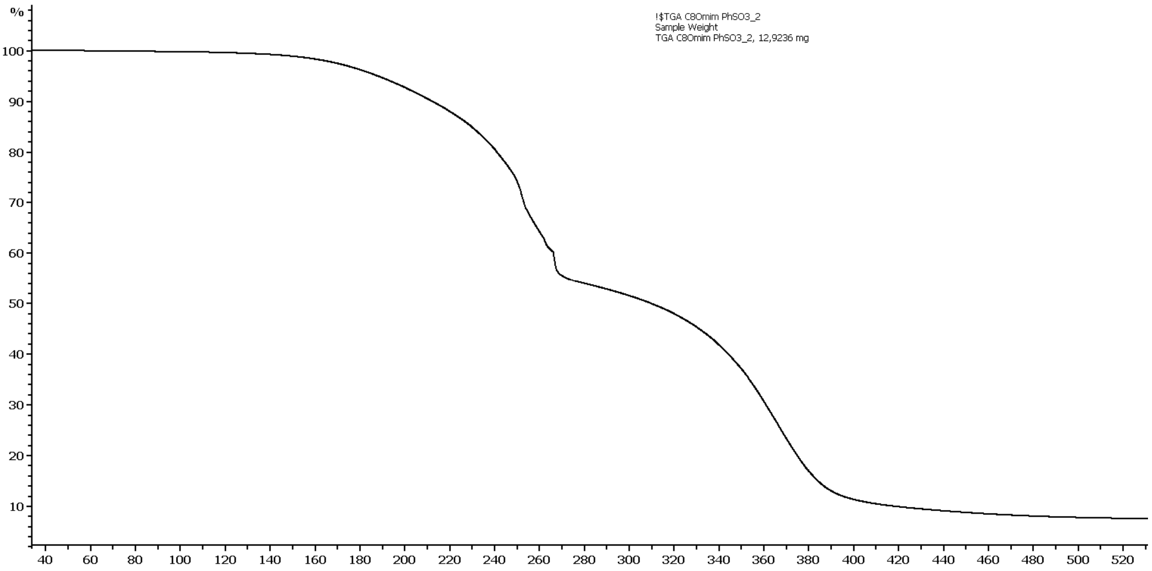

TGA curve for compound BE-2

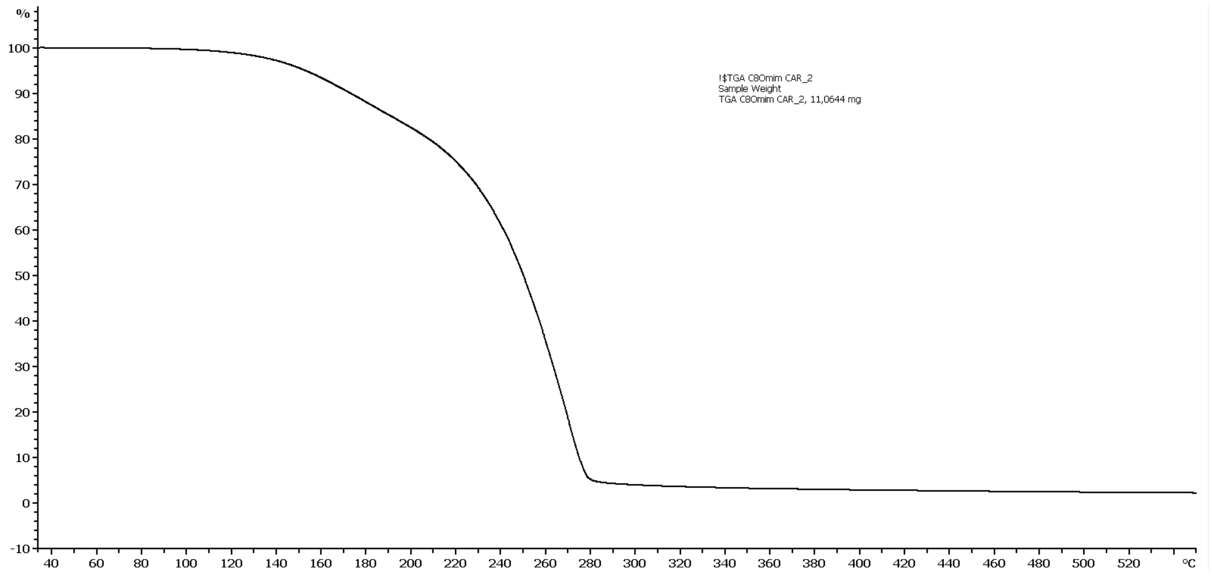

TGA curve for compound OR-3

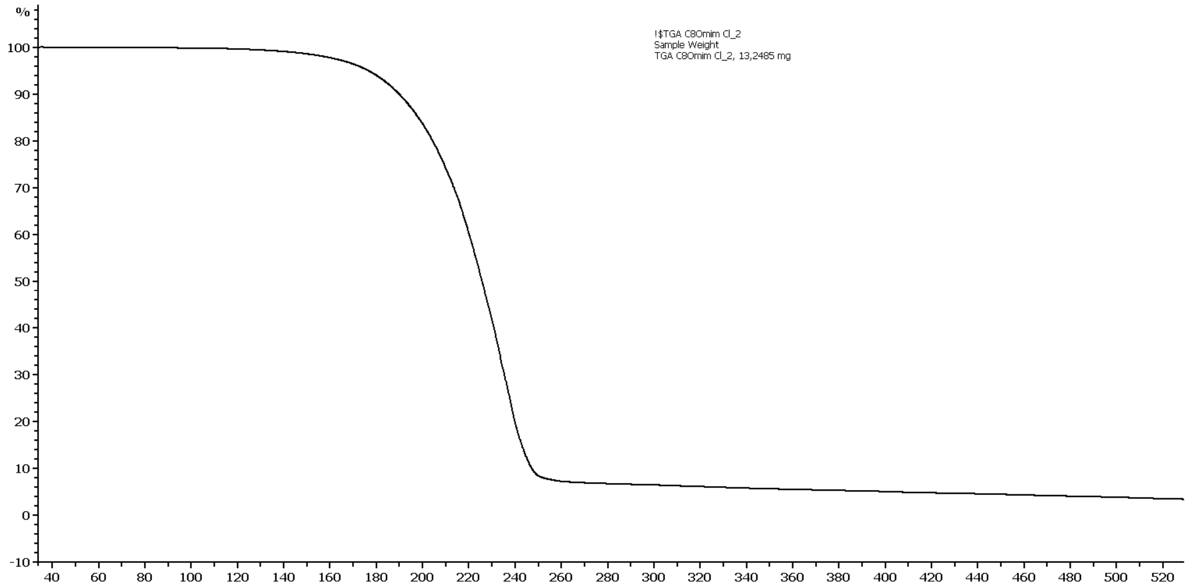

TGA curve for compound CL-4

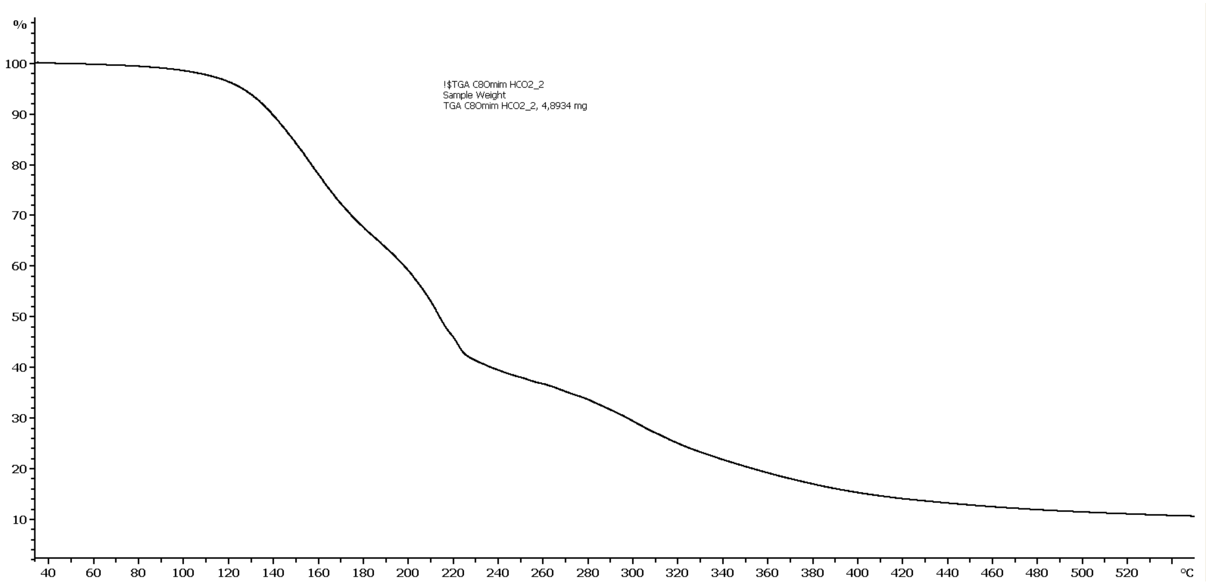

TGA curve for compound MR-5

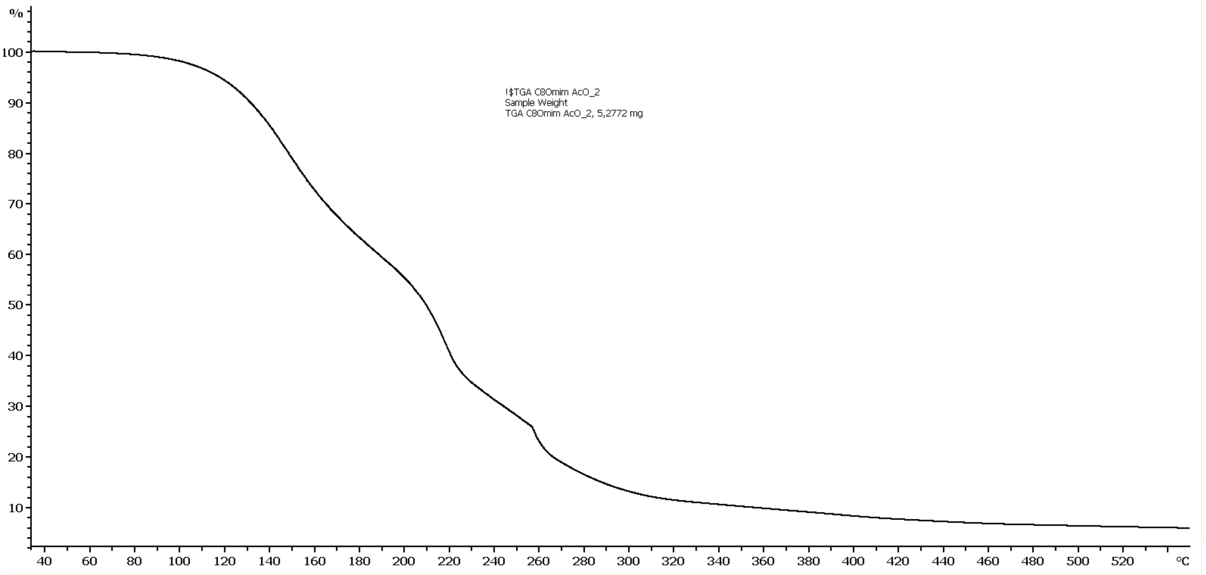

TGA curve for compound PR-6

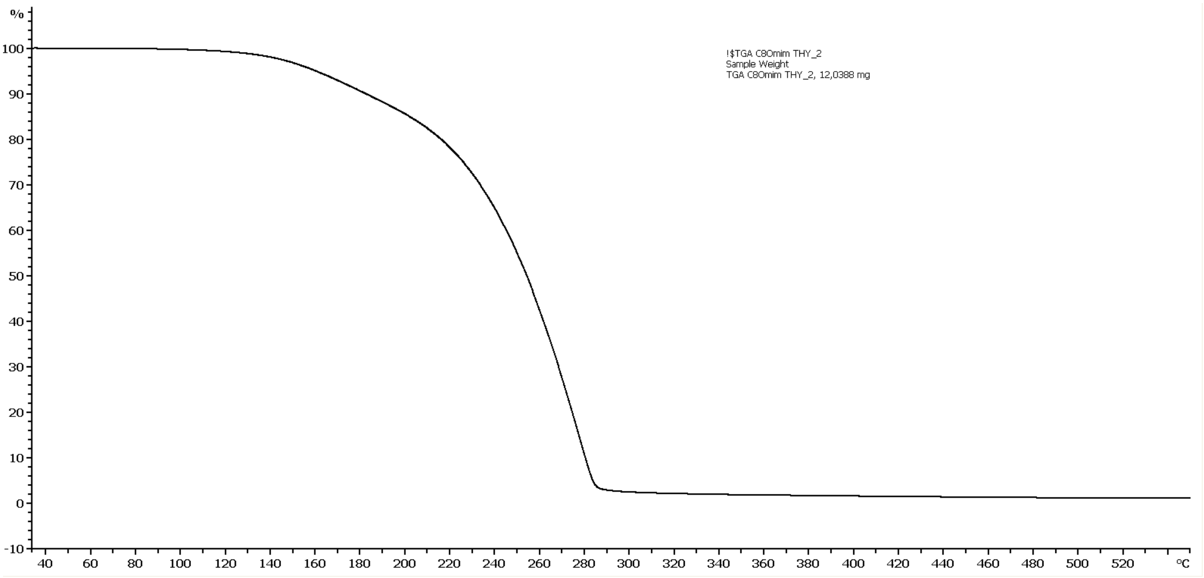

TGA curve for compound TY-7

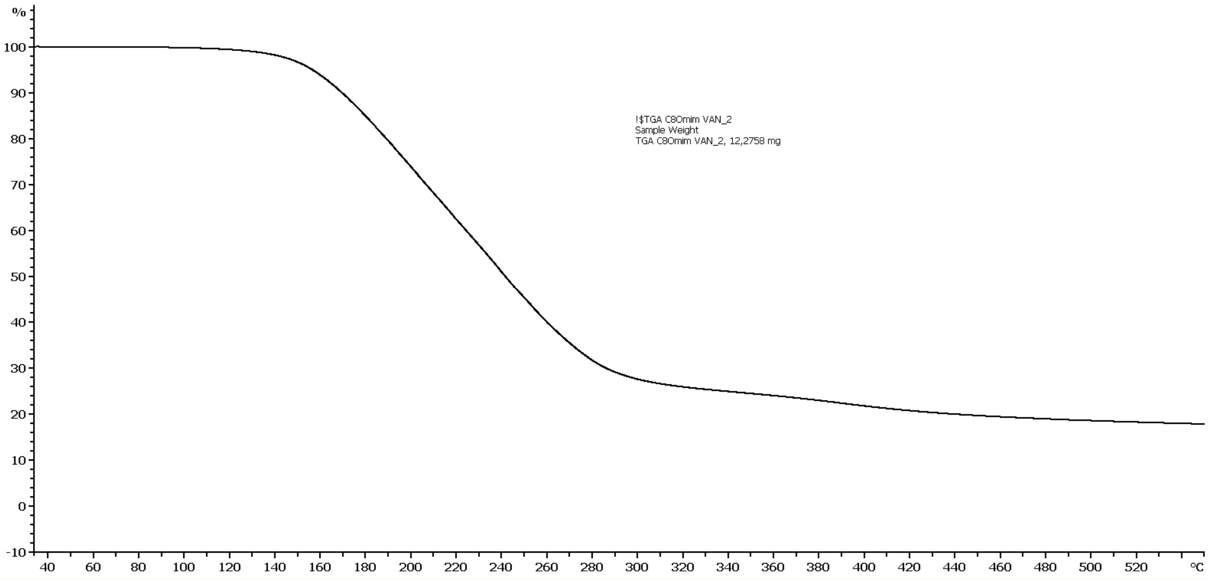

TGA curve for compound VA-8

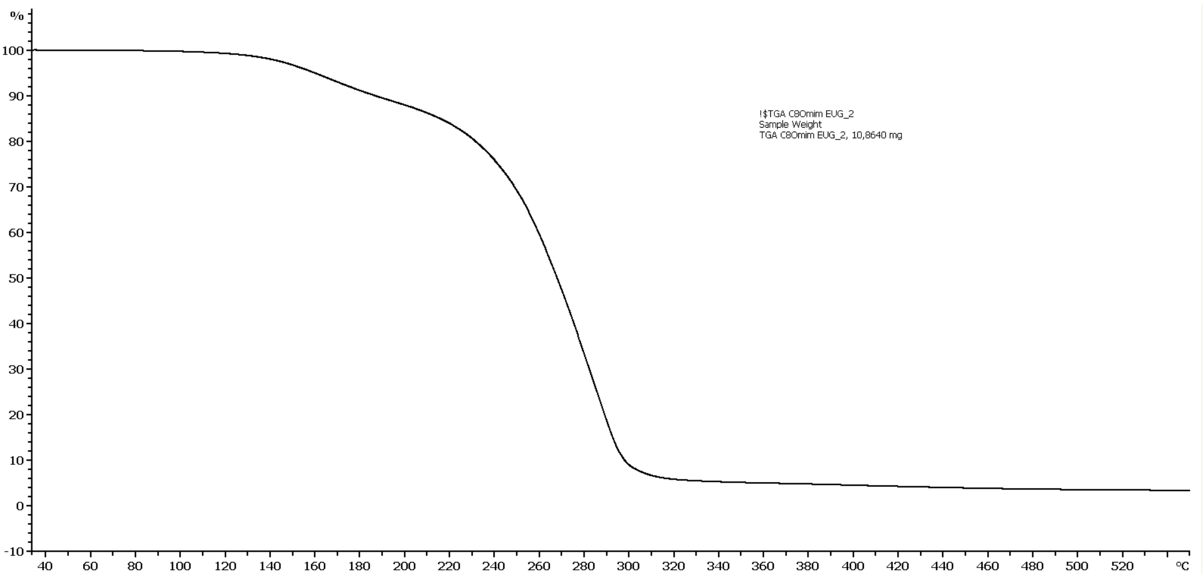

TGA curve for compound EV-9

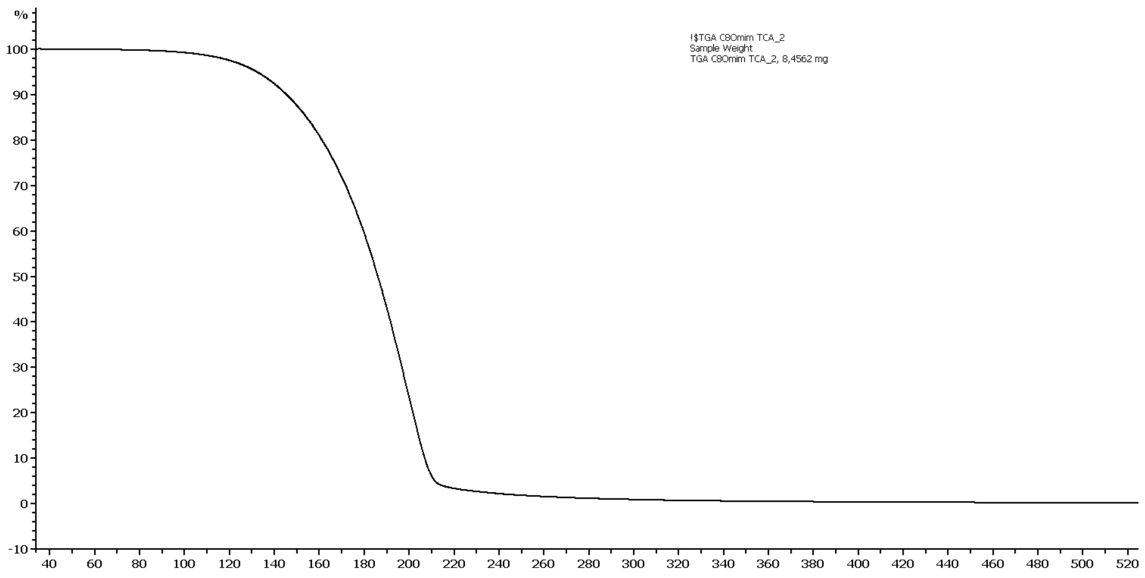

TGA curve for compound TR-10

Section S1.4. Elementary analysis

Table S3. Elementary analysis for obtained ionic liquids

Compound	Formula	calculated	obtained
FE-1	$C_{19}H_{30}N_2O_4S$	C(59.66%)<br>H(7.91%)<br>N(7.32%)<br>O(16.73%)<br>S(8.38%)	C(59.18%)<br>H(7.42%)<br>N(6.85%)<br>O(16.21%)<br>S(7.91%)
BE-2	C19H30N2O5S	C(57.26%)<br>H(7.59%)<br>N(7.03%)<br>O(20.07%)	C(57.69%)<br>H(7.96%)<br>N(7.44%)<br>O(20.45%)

		S(8.05%)	S(8.52%)
OR-3	$C_{25}H_{40}N_2O_4$	C(69.41%)<br>H(9.32%)<br>N(6.48%)<br>O(14.79%)	C(69.01%)<br>H(8.92%)<br>N(6.09%)<br>O(14.38%)
CL-4	$C_{13}H_{25}ClN_2O$	C(59.87%)<br>H(9.66%)<br>Cl(13.59%)<br>N(10.74%)<br>O(6.13%)	C(59.25%)<br>H(9.13%)<br>Cl(13.02%)<br>N(10.20%)<br>O(5.73%)
MR-5	$C_{14}H_{26}N_2O_3$	C(62.19%)<br>H(9.69%)<br>N(10.36%)<br>O(17.75%)	C(62.49%)<br>H(9.98%)<br>N(10.66%)<br>O(18.09%)
PR-6	$C_{15}H_{28}N_2O_3$	C(63.35%)<br>H(9.92%)<br>N(9.85%)<br>O(16.88%)	C(63.02%)<br>H(9.66%)<br>N(9.23%)<br>O(16.26%)
TY-7	$C_{25}H_{40}N_2O_4$	C(69.41%)<br>H(9.32%)<br>N(6.48%)<br>O(14.79%)	C(69.90%)<br>H(9.78%)<br>N(6.96%)<br>O(15.26%)
VA-8	$C_{23}H_{34}N_2O_6$	C(63.57%)<br>H(7.89%)<br>N(6.45%)<br>O(22.09%)	C(63.05%)<br>H(7.36%)<br>N(5.99%)<br>O(21.66%)
EV-9	$C_{25}H_{38}N_2O_5$	C(67.24%)<br>H(8.58%)<br>N(6.27%)<br>O(17.91%)	C(67.81%)<br>H(8.93%)<br>N(6.81%)<br>O(18.42%)
TR-10	$C_{18}H_{34}N_2O_3$	C(66.22%)<br>H(10.50%)<br>N(8.58%)<br>O(14.70%)	C(66.44%)<br>H(10.93%)<br>N(8.99%)<br>O(15.07%)

Section S1.5. 1H and 13C-NMR spectroscopy

The novel group of imidazolium based ionic liquids was characterized by ^{1}H and ^{13}C-NMR spectroscopy. ^{1}H and ^{13}C NMR spectra were recorded at 25 °C using a 400 MHz (for 1H) and 100 MHz (for 13C) Bruker NMR spectrometer.

1-methyl-3-octyloxymetyloimidazolium 4-hydroxybenzenesulfonate (FE-1):

1H NMR (DMSO-d6) δ ppm = 0.84-0.87 (t, J=6.85 Hz, 3H); 1.23-1.27 (m, 12H); 1.47 (m, 2H); 3.45-3.48 (m, 3H); 6.8-6.70 (d, 2H); 7.42-7.44 (d, 2H); 7.79 (s, 1H); 7.86 (s, 1H); 9.38 (s, 1H).

13C NMR (DMSO-d6) δ ppm = 13.97; 22.16; 25.37; 28.77; 28.79; 29.03; 31.36; 35.91; 69.18; 78.09; 114.06; 121.87; 124.01; 127.02; 137.26; 139.04; 157.72.

1-methyl-3-octyloxymethylimidazolium benzenesulfonate (BE-2):

1H NMR (DMSO-d6) δ ppm = 0.84-0.87 (t, J=6.8 Hz, 3H); 1.23-1.27 (m, 12H); 1.47 (m, 2H); 3,89 (s, 3H); 5.56 (s, 2H); 7.31-7.64 (m, 5H); 7.78 (s, 1H); 7.86 (s, 1H); 9.34 (s, 1H).

13C NMR (DMSO-d6) δ ppm = 13.99; 22.17; 25.38; 28.71; 28.77; 29.05; 31.36; 35.95; 69.19; 78.12; 121.90; 124.03; 125.46; 127.70; 128.53; 137.20; 148.13.

1-methyl-3-octyloxymethylimidazolium carvacroloxyacetate (OR-3):

1H NMR (DMSO-d6) δ ppm = 0.84-0.87 (t, J=6.85 Hz, 3H); 1.12-1.14 (d, 6H); 1.22-1.27 (m, 12H); 1.46 (m, 2H); 2.74 (m, 1H); 3.87 (s, 3H); 5.57 (d, 2H); 6.59-6.61 (m, 2H); 6.94-6.96 (m, 1H); 7.83 (s, 1H); 7.91 (s, 1H); 9.90 (s, 1H).

13C NMR (DMSO-d6) δ ppm = 13.95; 1.92; 22.16; 23.96; 25.40; 28.74; 28.77; 29.03; 31.36; 35.91; 68.08; 6.08; 77.93; 109.72; 116.52; 121.87; 122.61; 123.99; 127.02; 137.99; 146.76; 157.18; 170.61.

1-methyl-3-octyloxymethylimidazolium chloride (CL-4):

1H NMR (DMSO-d6) δ ppm = 0.84-0.87 (t, J=6.85 Hz, 3H); 1.23-1.28 (m, 12H); 1.49 (m, 2H); 3.50-3.53 (t, J=6.5 Hz, 3H); 5.69 (s, 2H); 7.85-7.86 (t, J=1.75 Hz, 1H); 8.01-8.02 (t, J=1.8Hz,1H); 9.82 (s, 1H).

13C NMR (DMSO-d6) δ ppm = 13.89; 22.09; 25.34; 28.70; 28.74; 28.96; 31.29; 35.87; 69.11; 77.90; 121.85; 123.93; 137.39.

1-methyl-3-octyloxymethylimidazolium formate (MR-5):

1H NMR (DMSO-d6) δ ppm = 0.84-0.87 (t, J=6.85 Hz, 3H); 1.23-1.28 (m, 12H); 1.49 (m, 2H); 3.42-3.49 (m, 3H); 5.52 (s, 2H); 7.84-7.85 (t, J=1.7 Hz, 1H); 7.91-7.92 (t, J=1.7Hz, 1H); 9.53 (s, 1H); 9.72 (s, 1H).

13C NMR (DMSO-d6) δ ppm = 13.92; 22.13; 25.37; 28.71; 28.74; 29.00; 31.33; 35.87; 69.14; 78.02; 121.87; 124.02; 137.64; 165.65.

1-methyl-3-octyloxymethylimidazolium propioniate (PR-6):

1H NMR (DMSO-d6) δ ppm = 0.84-0.87 (t, J=6.85 Hz, 3H); 1.23-1.27 (m, 12H); 1.49 (m, 2H); 1.67 (s, 3H); 3.93 (m, 3H); 4.86 (s, 2H); 5.62 (s, 2H); 7.84 (s, 1H); 7.91(s, 1H); 9.87 (s, 1H).

13C NMR (DMSO-d6) δ ppm = 13.90; 18.54, 22.12; 25.36; 28.70; 28.73; 28.75; 28.99; 31.32; 35.81; 55.90; 69.09; 77.98; 121.83; 123.98; 137.92; 173.64.

1-methyl-3-octyloxymethylimidazolium thymoloxyacetate (TY-7):

1H NMR (DMSO-d6) δ ppm = 0.84-0.87 (t, J=6.85 Hz, 3H); 1.12-1.14 (d, 6H); 1.22-1.27 (m, 12H); 1.46 (m, 2H); 2.74 (m, 1H); 3.87 (s, 3H); 5.57 (d, 2H); 6.59-6.61 (m, 2H); 6.94-6.96 (m, 1H); 7.83 (s, 1H); 7.91 (s, 1H); 9.90 (s, 1H).

13C NMR (DMSO-d6) δ ppm = 13.95; 15.92; 22.16; 23.96; 25.40; 28.74; 28.77; 29.03; 31.36; 35.91; 68.08; 69.08; 77.93; 109.72; 116.52; 121.87; 122.61; 123.99; 12.,02; 137.99; 146.76; 157.18; 170.61.

1-methyl-3-octyloxymethylimidazolium vanillinoxyacetate (VA-8):

1H NMR (DMSO-d6) δ ppm = 0.84-0.87 (t, J=6.7 Hz, 3H); 1.21 (m, 12H); 1.47 (m, 2H); 3.50-3.53 (m, 3H); 3.82-3.93 (m, 3H); 4.36 (s, 2H); 5.59 (s, 2H); 6.74-6.76 (m, 1H); 7.36-7.89 (m, 2H); 9.57 (s, 1H); 9.81 (s, 1H).

13C NMR (DMSO-d6) δ ppm = 13.98; 22.16; 25.37; 28.76; 29.01 29.04; 31.35; 35.95; 55.11; 69.20; 77.16; 109.62; 116.04; 121.91; 124.05; 124.18; 127.98; 147.95; 149.76; 160.86; 189.19.

1-methyl-3-octyloxymethylimidazolium eugenoloxyacetate (EV-9):

1H NMR (DMSO-d6) δ ppm = 0.83-0.87 (t, J=6.9 Hz, 3H), 1.21-1.27 (m, 12H), 1.45 (s, 2H), 3.25-3.27 (d, 2H), 3.69 (s, 3H), 3.86 (s, 3H), 4.15 (s, 2H), 4.99-5.08 (m, 2H), 5.57 (s, 2H); 5.89-5.95 (m, 1H); 6.57-6.59 (m, 1H) 6.65-6.70 (m, 2H); 7.81 (s, 1H); 7.88 (s, 1H); 9.90 (s, 1H).

13C NMR (DMSO-d6) δ ppm = 13.95; 22.15; 25.40; 28.76; 28.80; 29.02; 29.05; 31.35; 35.71; 35.72; 55.29; 55.21; 68.07; 69.08; 77.95; 112.09; 112.50; 115.24; 119.93; 121.78; 123.95; 131.01; 138.10; 146.90; 148.39; 170.53.

1-methyl-3-octyloxymethylimidazolium trimethylacetate (TR-10):

1H NMR (DMSO-d6) δ ppm = 0.84-0.87 (t, J=6.9 Hz, 3H); 1.00 (s, 9H), 1.23-1.27 (m, 12H); 1.48 (m, 2H); 3.94 (m, 3H); 5.65 (s, 2H); 7.86-7.87 (t, J=1.7 Hz, 1H); 7.93-7.94 (t, J=1.7 Hz, 1H); 10.29 (s, 1H).

13C NMR (DMSO-d6) δ ppm = 13.92; 22.16; 25.37; 28.68; 28.73; 28.77; 28.80; 29.02; 29.06; 31.32; 35.74; 69.01; 77.89; 121.84; 123.99; 138.62; 180.84.

1-methyl-3-octyloxymetyloimidazolium 4-hydroxybenzenesulfonate (FE-1):

This report was created by ACD/NMR Processor Academic Edition. For more information go to www.acdlabs.com/nmrproc/

2021-11-16 19:55:24

Acquisition Time (sec)	5.0000	*Date*	Nov 15 2021	*Date Stamp*	Nov 15 2021		
File Name	d:\Users\Michal\Desktop\MW-1.fid\fid	*Frequency (MHz)*	399.91	*Nucleus*	1H	*Number of Transients*	64
Original Points Count	37879	*Points Count*	65536	*Pulse Sequence*	s2pul	*Receiver Gain*	18.00
Solvent	DMSO-d6	*Spectrum Offset (Hz)*	3419.2983	*Spectrum Type*	STANDARD	*Sweep Width (Hz)*	7575.76
Temperature (degree C)	25.000						

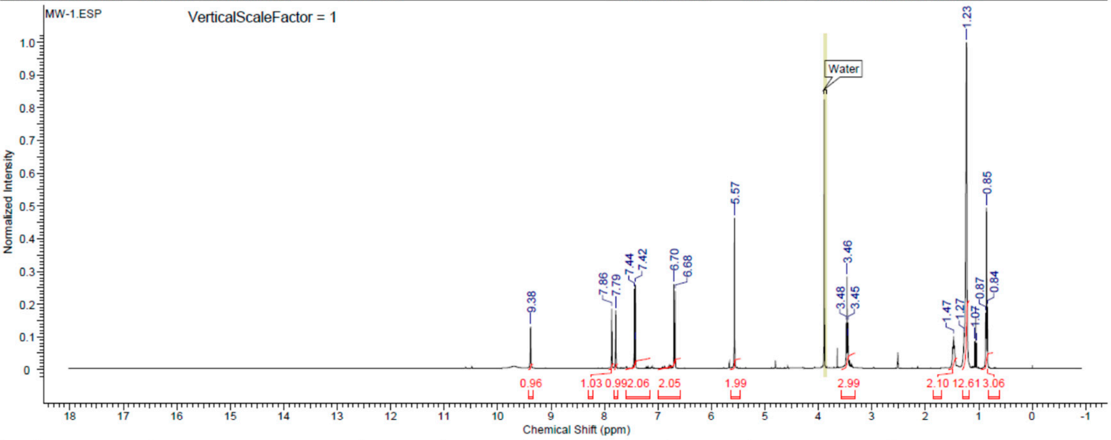

No.	(ppm)	Annotation	Layer No.	Created By	Created At	Modified By	Modified At
1	[3.85 .. 3.90]	Water	1	Michal	Wt 2021-11-16 16:25:41		

No.	(ppm)	(Hz)	Height	No.	(ppm)	(Hz)	Height	No.	(ppm)	(Hz)	Height	No.	(ppm)	(Hz)	Height	No.	(ppm)	(Hz)	Height
1	0.84	334.7	0.1922	6	1.07	429.9	0.0856	11	3.46	1382.0	0.0958	16	6.68	2672.5	0.0841	21	7.79	3115.4	0.1810
2	0.85	341.8	0.4948	7	1.23	491.4	1.0000	12	3.46	1384.8	0.2833	17	6.70	2679.2	0.2604	22	7.86	3144.9	0.1855
3	0.87	348.4	0.1735	8	1.27	509.0	0.1079	13	3.48	1391.3	0.1439	18	7.42	2967.8	0.2593	23	9.38	3752.0	0.1310
4	1.04	415.9	0.0812	9	1.47	587.8	0.1013	14	5.57	2226.8	0.4637	19	7.43	2969.8	0.0847				
5	1.06	422.9	0.1626	10	3.45	1378.2	0.1443	15	6.68	2670.5	0.2397	20	7.44	2976.5	0.2455				

This report was created by ACD/NMR Processor Academic Edition. For more information go to www.acdlabs.com/nmrproc/

2021-11-16 19:58:33

Acquisition Time (sec)	1.0000	*Date*	Nov 15 2021	*Date Stamp*	Nov 15 2021		
File Name	d:\Users\Michal\Desktop\MW-1-C13.fid\fid	*Frequency (MHz)*	100.57	*Nucleus*	13C	*Number of Transients*	1008
Original Points Count	22322	*Points Count*	32768	*Pulse Sequence*	s2pul	*Receiver Gain*	58.00
Solvent	DMSO-d6	*Spectrum Offset (Hz)*	9728.0635	*Spectrum Type*	STANDARD	*Sweep Width (Hz)*	22321.43
Temperature (degree C)	25.000						

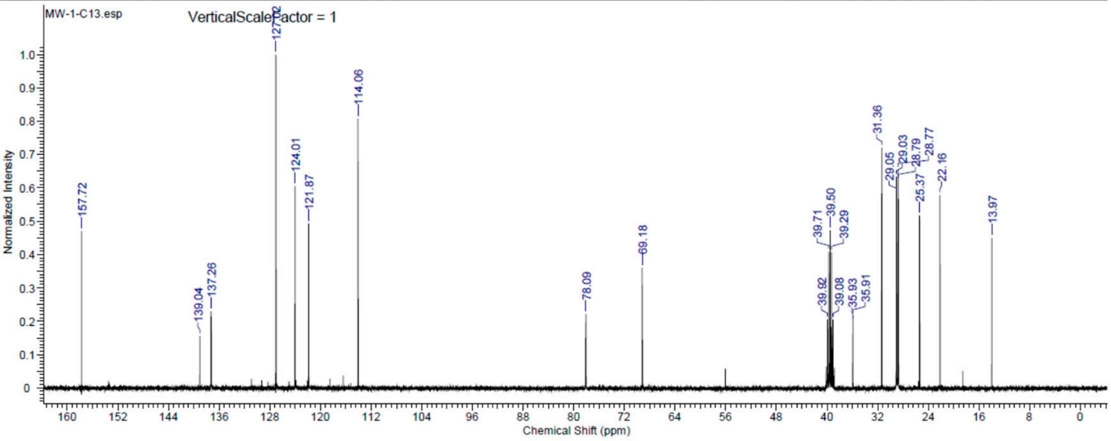

No.	(ppm)	(Hz)	Height	No.	(ppm)	(Hz)	Height	No.	(ppm)	(Hz)	Height	No.	(ppm)	(Hz)	Height	No.	(ppm)	(Hz)	Height
1	13.97	1404.6	0.4503	6	28.79	2895.1	0.6221	11	35.93	3613.1	0.2008	16	39.92	4014.4	0.2054	21	124.01	12471.0	0.6050
2	22.16	2228.9	0.5779	7	29.03	2919.0	0.6330	12	39.08	3930.6	0.2047	17	69.18	6957.2	0.3611	22	127.02	12773.4	1.0000
3	25.37	2551.8	0.5169	8	29.05	2921.7	0.5809	13	39.29	3951.7	0.4061	18	78.09	7853.0	0.2210	23	137.26	13804.1	0.2291
4	28.71	2887.6	0.5666	9	31.36	3153.3	0.7199	14	39.50	3972.1	0.4727	19	114.06	11471.0	0.8079	24	139.04	13982.6	0.1554
5	28.77	2893.1	0.6547	10	35.91	3611.8	0.2156	15	39.71	3993.2	0.4084	20	121.87	12255.7	0.4934	25	157.72	15860.7	0.4706

1-methyl-3-octyloxymethylimidazolium benzenesulfonate (BE-2):

This report was created by ACD/NMR Processor Academic Edition. For more information go to www.acdlabs.com/nmrproc/

2021-11-17 18:44:00

Acquisition Time (sec)	5.0001	*Date*	Nov 17 2021			*Date Stamp*	Nov 17 2021		
File Name	d:\Users\Michal\Desktop\MW-2.fid\fid			*Frequency (MHz)*	399.91	*Nucleus*	1H	*Number of Transients*	40
Original Points Count	30941	*Points Count*	32768	*Pulse Sequence*	s2pul	*Receiver Gain*	18.00	*Solvent*	DMSO-d6
Spectrum Offset (Hz)	2743.7021	*Spectrum Type*	STANDARD			*Sweep Width (Hz)*	6188.12	*Temperature (degree C)*	25.000

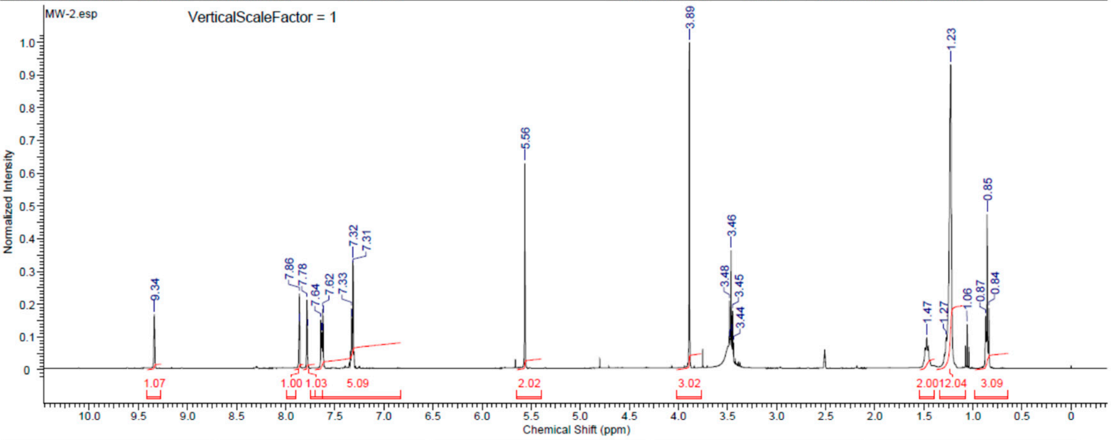

No.	(ppm)	(Hz)	Height
1	0.84	334.8	0.1880
2	0.85	341.8	0.4741
3	0.87	348.4	0.1645
4	1.06	423.2	0.1385
5	1.23	490.4	0.9323
6	1.27	508.9	0.0985
7	1.47	588.0	0.0990
8	3.44	1375.0	0.0828
9	3.45	1378.4	0.1804
10	3.46	1382.0	0.1108
11	3.46	1384.8	0.3646
12	3.47	1389.0	0.0935
13	3.48	1391.4	0.2109
14	3.89	1555.4	1.0000
15	5.56	2225.0	0.6307
16	7.31	2924.0	0.3188
17	7.32	2925.9	0.3341
18	7.32	2928.9	0.1558
19	7.33	2931.1	0.1876
20	7.62	3046.1	0.1652
21	7.62	3048.0	0.1853
22	7.63	3051.2	0.1141
23	7.63	3052.9	0.1126
24	7.64	3053.7	0.0978
25	7.64	3055.0	0.0899
26	7.64	3056.0	0.1524
27	7.78	3110.5	0.1256
28	7.78	3112.2	0.2137
29	7.79	3113.9	0.1391
30	7.86	3141.7	0.1401
31	7.86	3143.6	0.2323
32	7.87	3145.3	0.1375
33	9.34	3734.3	0.1693

This report was created by ACD/NMR Processor Academic Edition. For more information go to www.acdlabs.com/nmrproc/

2021-11-16 19:57:00

Acquisition Time (sec)	1.0000	*Date*	Nov 15 2021			*Date Stamp*	Nov 15 2021		
File Name	d:\Users\Michal\Desktop\MW-2-C13.fid\fid			*Frequency (MHz)*	100.57	*Nucleus*	13C	*Number of Transients*	944
Original Points Count	22322	*Points Count*	32768	*Pulse Sequence*	s2pul	*Receiver Gain*	58.00	*Solvent*	DMSO-d6
Spectrum Offset (Hz)	9729.0850	*Spectrum Type*	STANDARD			*Sweep Width (Hz)*	22321.43	*Temperature (degree C)*	25.000

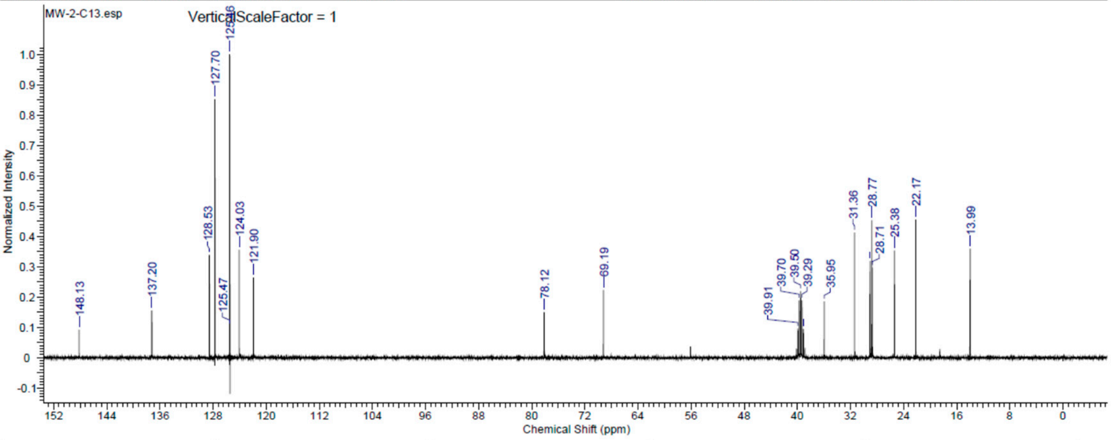

No.	(ppm)	(Hz)	Height
1	13.99	1407.0	0.3592
2	22.17	2229.2	0.4548
3	25.38	2552.1	0.3531
4	28.71	2887.3	0.2964
5	28.77	2893.4	0.4529
6	29.02	2918.6	0.2881
7	29.05	2921.3	0.3183
8	31.36	3153.6	0.4119
9	35.95	3615.5	0.1851
10	39.08	3930.2	0.0940
11	39.29	3951.3	0.1888
12	39.50	3972.5	0.2185
13	39.70	3992.9	0.1879
14	39.91	4014.0	0.0946
15	69.19	6958.2	0.2227
16	78.12	7856.1	0.1499
17	121.90	12258.8	0.2633
18	124.03	12472.7	0.3550
19	125.46	12617.1	1.0000
20	125.47	12618.5	0.1032
21	127.70	12841.9	0.8514
22	128.53	12925.7	0.3379
23	137.20	13797.7	0.1555
24	148.13	14896.5	0.0917

1-methyl-3-octyloxymethylimidazolium carvacroloxyacetate (OR-3):

This report was created by ACD/NMR Processor Academic Edition. For more information go to www.acdlabs.com/nmrproc/

2021-11-17 18:45:07

Acquisition Time (sec)	4.9999	*Date*	Nov 16 2021			*Date Stamp*	Nov 16 2021		
File Name	d:\Users\Michal\Desktop\MW-7.fid\fid			*Frequency (MHz)*	399.91	*Nucleus*	1H	*Number of Transients*	64
Original Points Count	32216	*Points Count*	32768	*Pulse Sequence*	s2pul	*Receiver Gain*	18.00	*Solvent*	DMSO-d6
Spectrum Offset (Hz)	2851.3411	*Spectrum Type*	STANDARD			*Sweep Width (Hz)*	6443.30	*Temperature (degree C)*	25.000

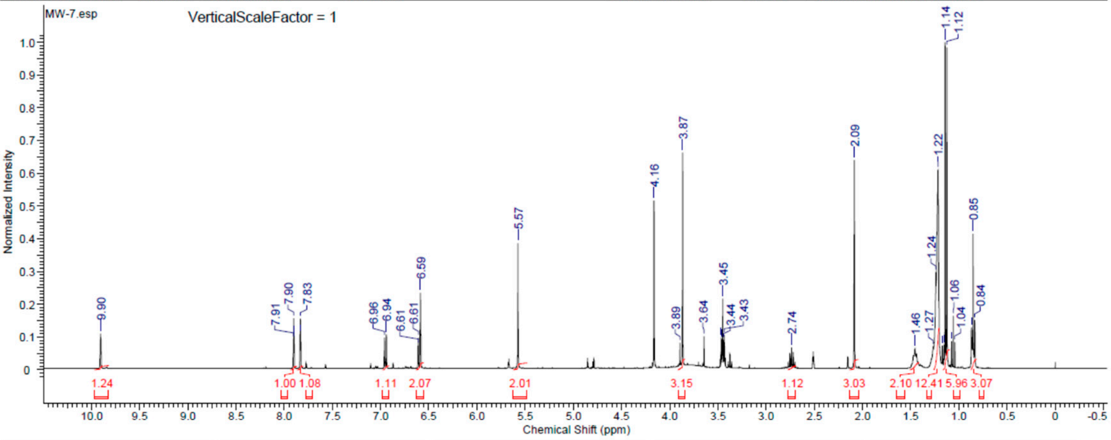

No.	(ppm)	(Hz)	Height	No.	(ppm)	(Hz)	Height	No.	(ppm)	(Hz)	Height	No.	(ppm)	(Hz)	Height	No.	(ppm)	(Hz)	Height
1	0.84	334.1	0.1511	9	1.15	461.3	0.0754	17	3.43	1373.1	0.1064	25	4.16	1664.7	0.5165	33	7.83	3131.8	0.1557
2	0.85	341.1	0.4144	10	1.17	468.2	0.0741	18	3.44	1376.8	0.0919	26	5.57	2229.5	0.3860	34	7.84	3133.6	0.1084
3	0.87	347.8	0.1293	11	1.22	486.6	0.6108	19	3.45	1379.6	0.2166	27	6.59	2634.2	0.2342	35	7.90	3157.8	0.1039
4	1.04	416.6	0.0844	12	1.24	495.9	0.2981	20	3.46	1383.7	0.0936	28	6.61	2642.0	0.0979	36	7.90	3159.6	0.1564
5	1.06	423.7	0.1646	13	1.27	506.9	0.0706	21	3.47	1386.3	0.0990	29	6.61	2643.6	0.0744	37	7.91	3161.3	0.0939
6	1.08	430.6	0.0864	14	1.46	582.4	0.0638	22	3.64	1457.7	0.1018	30	6.94	2775.5	0.1077	38	9.90	3960.9	0.1108
7	1.12	449.9	0.9842	15	2.09	833.9	0.6394	23	3.87	1546.3	0.6631	31	6.96	2783.0	0.0981				
8	1.14	456.8	1.0000	16	2.74	1094.1	0.0688	24	3.89	1556.8	0.0823	32	7.83	3130.3	0.0850				

This report was created by ACD/NMR Processor Academic Edition. For more information go to www.acdlabs.com/nmrproc/

2021-11-17 18:46:37

Acquisition Time (sec)	1.0000	*Date*	Nov 16 2021			*Date Stamp*	Nov 16 2021		
File Name	d:\Users\Michal\Desktop\MW-7-C13.fid\fid			*Frequency (MHz)*	100.57	*Nucleus*	13C	*Number of Transients*	1008
Original Points Count	22322	*Points Count*	32768	*Pulse Sequence*	s2pul	*Receiver Gain*	56.00	*Solvent*	DMSO-d6
Spectrum Offset (Hz)	9724.6572	*Spectrum Type*	STANDARD			*Sweep Width (Hz)*	22321.43	*Temperature (degree C)*	25.000

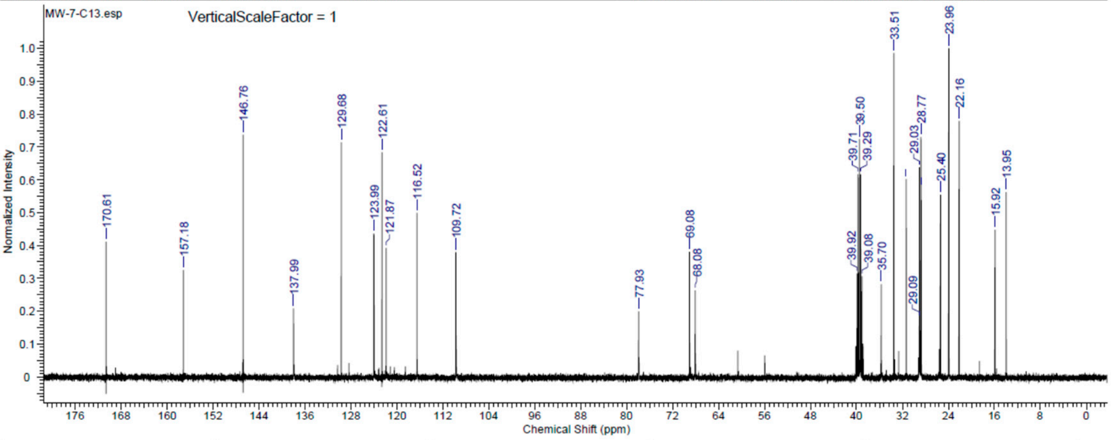

No.	(ppm)	(Hz)	Height	No.	(ppm)	(Hz)	Height	No.	(ppm)	(Hz)	Height	No.	(ppm)	(Hz)	Height	No.	(ppm)	(Hz)	Height
1	13.95	1403.3	0.5618	8	28.80	2896.5	0.5766	15	35.72	3592.7	0.2018	22	69.08	6947.0	0.3803	29	129.68	13041.8	0.7141
2	15.92	1600.8	0.4479	9	29.03	2919.0	0.6370	16	39.08	3930.6	0.3067	23	77.93	7836.7	0.1992	30	137.99	13877.0	0.2086
3	22.16	2228.9	0.7792	10	29.05	2921.7	0.5702	17	39.29	3951.0	0.6166	24	109.72	11033.6	0.3799	31	138.01	13879.1	0.1905
4	23.96	2409.4	1.0000	11	29.09	2925.1	0.1791	18	39.50	3972.1	0.7230	25	116.52	11717.6	0.4993	32	146.76	14759.2	0.7368
5	25.40	2554.5	0.5543	12	31.36	3153.3	0.6020	19	39.71	3993.2	0.6162	26	121.87	12256.4	0.3920	33	157.18	15806.9	0.3249
6	28.74	2890.4	0.5044	13	33.51	3369.9	0.9856	20	39.92	4014.4	0.3142	27	122.61	12330.0	0.6841	34	170.61	17157.8	0.4107
7	28.77	2893.8	0.7299	14	35.70	3590.6	0.2819	21	68.08	6846.2	0.2637	28	123.99	12469.6	0.4364				

1-methyl-3-octyloxymethylimidazolium chloride (CL-4):

This report was created by ACD/NMR Processor Academic Edition. For more information go to www.acdlabs.com/nmrproc/

2021-11-16 19:55:54

Acquisition Time (sec)	5.0000	*Date*	Nov 15 2021			*Date Stamp*	Nov 15 2021		
File Name	d:\Users\Michal\Desktop\MW-4.fid\fid			*Frequency (MHz)*	399.91	*Nucleus*	1H	*Number of Transients*	64
Original Points Count	37879	*Points Count*	65536	*Pulse Sequence*	s2pul	*Receiver Gain*	20.00	*Solvent*	DMSO-d6
Spectrum Offset (Hz)	3429.7598	*Spectrum Type*	STANDARD			*Sweep Width (Hz)*	7575.76	*Temperature (degree C)*	25.000

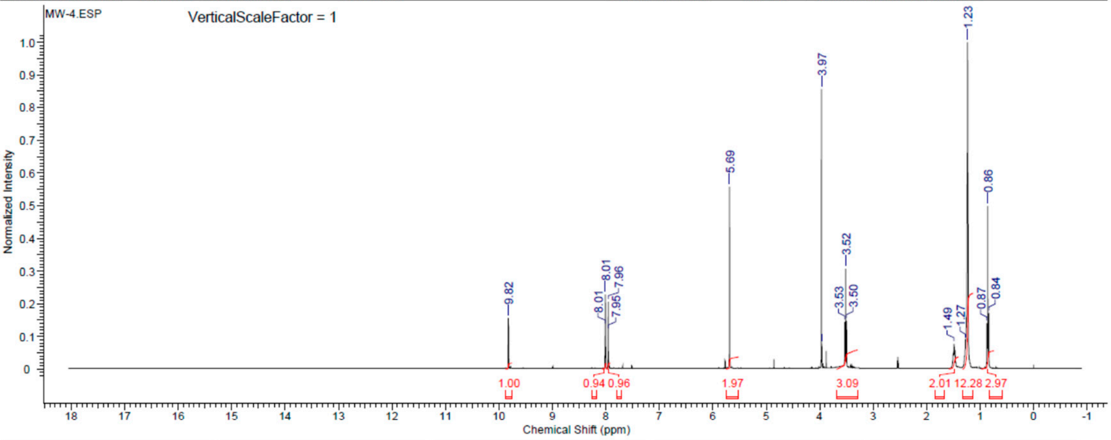

No.	(ppm)	(Hz)	Height
1	0.84	335.5	0.1711
2	0.86	342.5	0.4993
3	0.87	349.2	0.1407
4	1.23	493.8	1.0000
5	1.27	509.4	0.0879
6	1.49	594.9	0.0775
7	3.50	1399.6	0.1484
8	3.52	1406.0	0.3076
9	3.53	1412.6	0.1443
10	3.96	1584.7	0.0773
11	3.97	1587.2	0.8565
12	5.69	2274.2	0.5577
13	7.95	3180.1	0.1163
14	7.96	3181.9	0.2062
15	7.96	3183.6	0.1371
16	8.01	3201.3	0.1564
17	8.01	3203.0	0.2281
18	8.01	3204.9	0.1309
19	9.82	3928.2	0.1563
20	9.82	3928.6	0.1551

This report was created by ACD/NMR Processor Academic Edition. For more information go to www.acdlabs.com/nmrproc/

2021-11-16 19:57:40

Acquisition Time (sec)	1.0000	*Date*	Nov 15 2021			*Date Stamp*	Nov 15 2021		
File Name	d:\Users\Michal\Desktop\MW-4-C13-100sc.fid\fid			*Frequency (MHz)*	100.57	*Nucleus*	13C	*Number of Transients*	112
Original Points Count	22322	*Points Count*	32768	*Pulse Sequence*	s2pul	*Receiver Gain*	58.00	*Solvent*	DMSO-d6
Spectrum Offset (Hz)	9723.9766	*Spectrum Type*	STANDARD			*Sweep Width (Hz)*	22321.43	*Temperature (degree C)*	25.000

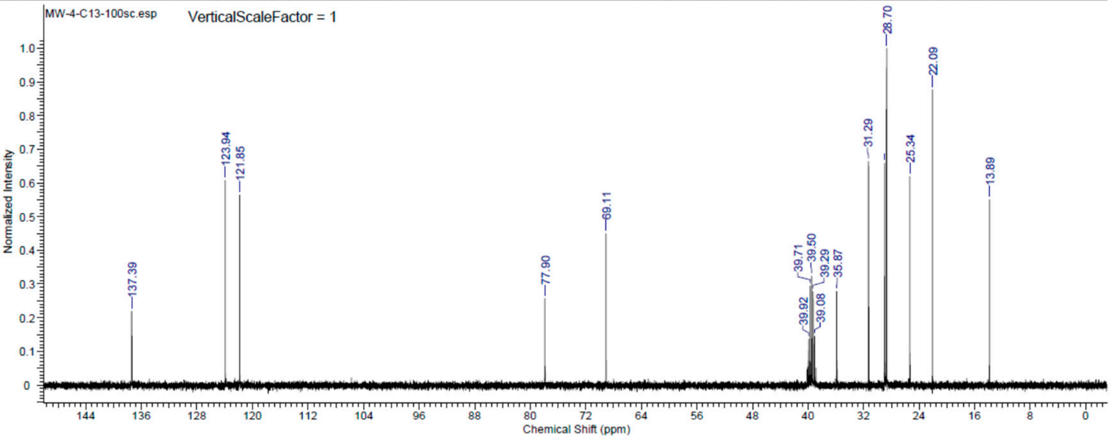

No.	(ppm)	(Hz)	Height
1	13.89	1396.4	0.5514
2	22.09	2221.4	0.8773
3	25.34	2548.4	0.6198
4	28.70	2886.3	1.0000
5	28.74	2890.4	0.4604
6	28.96	2912.8	0.6599
7	28.99	2915.6	0.5080
8	31.29	3146.5	0.6646
9	35.87	3607.7	0.2794
10	39.08	3929.9	0.1464
11	39.29	3951.0	0.2776
12	39.50	3972.8	0.3242
13	39.71	3993.2	0.2940
14	39.92	4014.4	0.1365
15	69.11	6949.7	0.4502
16	77.90	7833.9	0.2573
17	121.85	12254.4	0.5656
18	123.94	12464.2	0.6083
19	137.39	13816.4	0.2201

1-methyl-3-octyloxymethylimidazolium formate (MR-5):

This report was created by ACD/NMR Processor Academic Edition. For more information go to www.acdlabs.com/nmrproc/

2021-11-16 19:56:21

Acquisition Time (sec)	7.0000	Date	Nov 15 2021	Date Stamp	Nov 15 2021		
File Name	d:\Users\Michal\Desktop\MW-5.fid\fid	Frequency (MHz)	399.91	Nucleus	1H	Number of Transients	64
Original Points Count	45103	Points Count	65536	Pulse Sequence	s2pul	Receiver Gain	22.00
Solvent	DMSO-d6	Spectrum Offset (Hz)	2856.2053	Spectrum Type	STANDARD	Sweep Width (Hz)	6443.30
Temperature (degree C)	25.000						

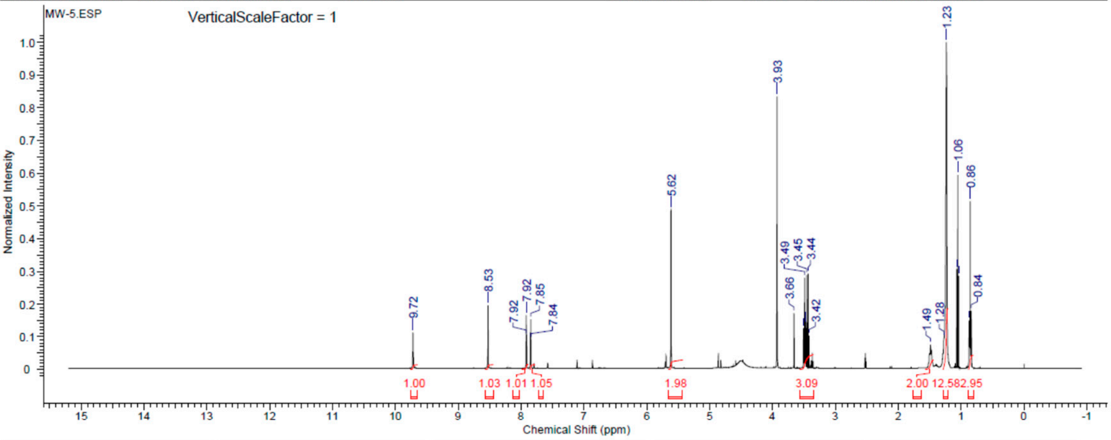

No.	(ppm)	(Hz)	Height
1	0.84	335.7	0.1790
2	0.86	342.7	0.5148
3	0.87	349.4	0.1496
4	1.04	415.1	0.2854
5	1.06	422.1	0.5945
6	1.07	429.1	0.3067
7	1.23	493.9	1.0000
8	1.28	509.9	0.0971
9	1.49	595.1	0.0761
10	3.42	1367.7	0.1038
11	3.44	1374.7	0.2937
12	3.45	1381.7	0.2891
13	3.47	1388.8	0.1368
14	3.48	1389.8	0.1466
15	3.49	1396.4	0.2808
16	3.51	1402.9	0.1266
17	3.66	1463.3	0.1723
18	3.93	1571.6	0.8349
19	5.62	2246.1	0.4873
20	7.84	3136.8	0.0907
21	7.85	3138.5	0.1532
22	7.85	3140.2	0.1130
23	7.91	3164.3	0.1067
24	7.92	3166.1	0.1655
25	7.92	3167.7	0.1012
26	8.53	3410.1	0.1958
27	9.72	3887.6	0.1134

This report was created by ACD/NMR Processor Academic Edition. For more information go to www.acdlabs.com/nmrproc/

2021-11-16 19:58:11

Acquisition Time (sec)	1.0000	Date	Nov 15 2021	Date Stamp	Nov 15 2021		
File Name	d:\Users\Michal\Desktop\MW-5-C13.fid\fid	Frequency (MHz)	100.57	Nucleus	13C	Number of Transients	800
Original Points Count	22322	Points Count	32768	Pulse Sequence	s2pul	Receiver Gain	56.00
Solvent	DMSO-d6	Spectrum Offset (Hz)	9723.9766	Spectrum Type	STANDARD	Sweep Width (Hz)	22321.43
Temperature (degree C)	25.000						

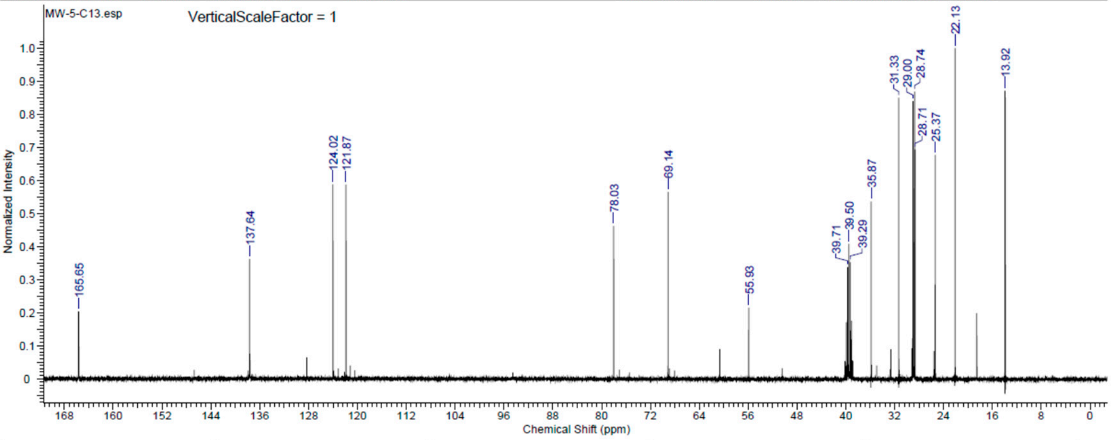

No.	(ppm)	(Hz)	Height
1	13.92	1399.8	0.8719
2	22.13	2225.5	1.0000
3	25.37	2551.1	0.6765
4	28.71	2886.9	0.6936
5	28.74	2890.4	0.8689
6	28.76	2892.4	0.7821
7	29.00	2916.2	0.8394
8	29.03	2919.0	0.7814
9	31.33	3150.6	0.8498
10	35.87	3607.0	0.5361
11	39.29	3951.7	0.3531
12	39.50	3972.8	0.4079
13	39.71	3993.9	0.3380
14	55.93	5624.8	0.2145
15	69.14	6953.1	0.5650
16	78.03	7846.9	0.4623
17	121.87	12255.7	0.5875
18	124.02	12471.7	0.5880
19	137.64	13842.3	0.3624
20	165.65	16659.1	0.2037

1-methyl-3-octyloxymethylimidazolium propioniate (PR-6):

This report was created by ACD/NMR Processor Academic Edition. For more information go to www.acdlabs.com/nmrproc/

2021-11-17 18:44:34

Acquisition Time (sec)	4.9999	*Date*	Nov 16 2021			*Date Stamp*	Nov 16 2021				
File Name	d:\Users\Michal\Desktop\MW-6.fid\fid			*Frequency (MHz)*	399.91	*Nucleus*	1H	*Number of Transients*	64		
Original Points Count	32216	*Points Count*	32768	*Pulse Sequence*	s2pul	*Receiver Gain*	56.00	*Solvent*	DMSO-d6		
Spectrum Offset (Hz)	2855.0256	*Spectrum Type*	STANDARD			*Sweep Width (Hz)*	6443.30	*Temperature (degree C)*	25.000		

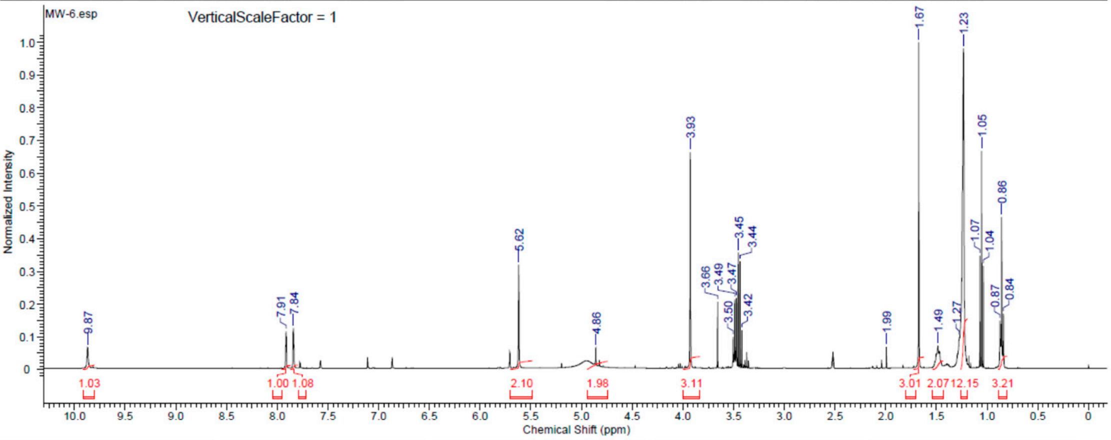

No.	(ppm)	(Hz)	Height	No.	(ppm)	(Hz)	Height	No.	(ppm)	(Hz)	Height	No.	(ppm)	(Hz)	Height	No.	(ppm)	(Hz)	Height
1	0.84	335.6	0.1698	6	1.07	428.6	0.3482	11	1.99	797.1	0.0695	16	3.49	1394.9	0.2146	21	5.62	2246.9	0.3208
2	0.86	342.7	0.4658	7	1.23	493.7	0.9805	12	3.42	1367.3	0.1208	17	3.50	1401.4	0.0993	22	7.84	3134.9	0.1264
3	0.87	349.3	0.1487	8	1.27	509.8	0.0981	13	3.44	1374.2	0.3301	18	3.66	1462.7	0.2067	23	7.91	3163.8	0.1159
4	1.04	414.6	0.3166	9	1.49	594.2	0.0729	14	3.45	1381.3	0.3575	19	3.93	1570.5	0.6637	24	9.87	3946.5	0.0684
5	1.05	421.5	0.6676	10	1.67	669.5	1.0000	15	3.47	1388.2	0.2181	20	4.86	1942.9	0.0670				

This report was created by ACD/NMR Processor Academic Edition. For more information go to www.acdlabs.com/nmrproc/

2021-11-17 18:45:55

Acquisition Time (sec)	1.0000	*Date*	Nov 16 2021			*Date Stamp*	Nov 16 2021				
File Name	d:\Users\Michal\Desktop\MW-6-C13.fid\fid			*Frequency (MHz)*	100.57	*Nucleus*	13C	*Number of Transients*	1008		
Original Points Count	22322	*Points Count*	32768	*Pulse Sequence*	s2pul	*Receiver Gain*	56.00	*Solvent*	DMSO-d6		
Spectrum Offset (Hz)	9722.9541	*Spectrum Type*	STANDARD			*Sweep Width (Hz)*	22321.43	*Temperature (degree C)*	25.000		

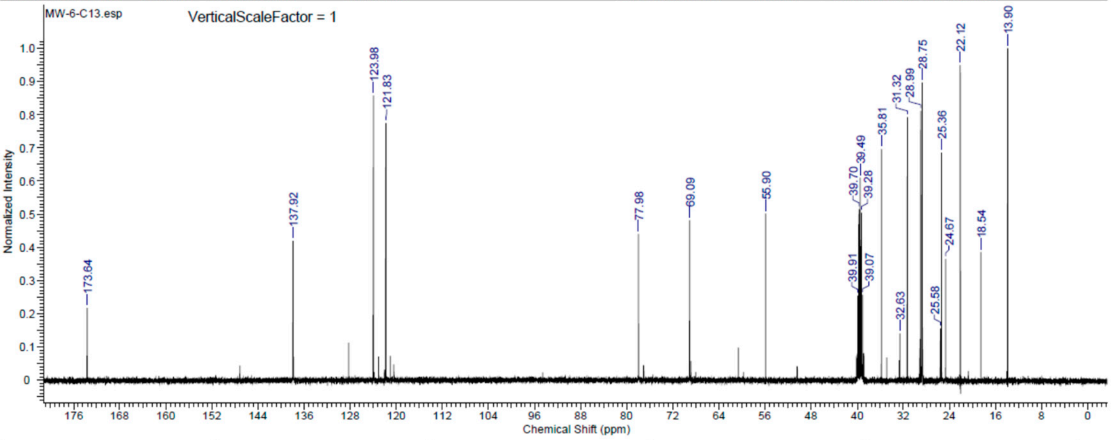

No.	(ppm)	(Hz)	Height	No.	(ppm)	(Hz)	Height	No.	(ppm)	(Hz)	Height	No.	(ppm)	(Hz)	Height	No.	(ppm)	(Hz)	Height
1	13.90	1398.1	1.0000	7	25.58	2572.6	0.1564	13	29.05	2921.3	0.2286	19	39.49	3971.8	0.6097	25	121.83	12252.0	0.7747
2	18.54	1864.8	0.3854	8	28.70	2886.6	0.8958	14	31.32	3149.6	0.7926	20	39.70	3992.9	0.5152	26	123.98	12468.6	0.8581
3	22.12	2224.5	0.9493	9	28.73	2889.3	0.8685	15	32.63	3281.0	0.1399	21	39.91	4014.0	0.2550	27	137.92	13869.9	0.4201
4	24.67	2480.6	0.3648	10	28.75	2891.4	0.8975	16	35.81	3601.2	0.6958	22	55.90	5621.7	0.5012	28	173.64	17462.6	0.2167
5	25.36	2550.1	0.6851	11	28.99	2915.2	0.8110	17	39.07	3929.5	0.2571	23	69.09	6948.0	0.4822				
6	25.38	2552.8	0.1214	12	29.02	2917.9	0.7544	18	39.28	3950.7	0.5044	24	77.98	7841.8	0.4405				

1-methyl-3-octyloxymethylimidazolium thymoloxyacetate (TY-7):

This report was created by ACD/NMR Processor Academic Edition. For more information go to www.acdlabs.com/nmrproc/

2021-11-17 18:45:07

Acquisition Time (sec)	4.9999	*Date*	Nov 16 2021		*Date Stamp*	Nov 16 2021	
File Name	d:\Users\Michal\Desktop\MW-7.fid\fid	*Frequency (MHz)*	399.91	*Nucleus*	1H	*Number of Transients*	64
Original Points Count	32216	*Points Count* 32768	*Pulse Sequence* s2pul	*Receiver Gain*	18.00	*Solvent*	DMSO-d6
Spectrum Offset (Hz)	2851.3411	*Spectrum Type*	STANDARD	*Sweep Width (Hz)*	6443.30	*Temperature (degree C)*	25.000

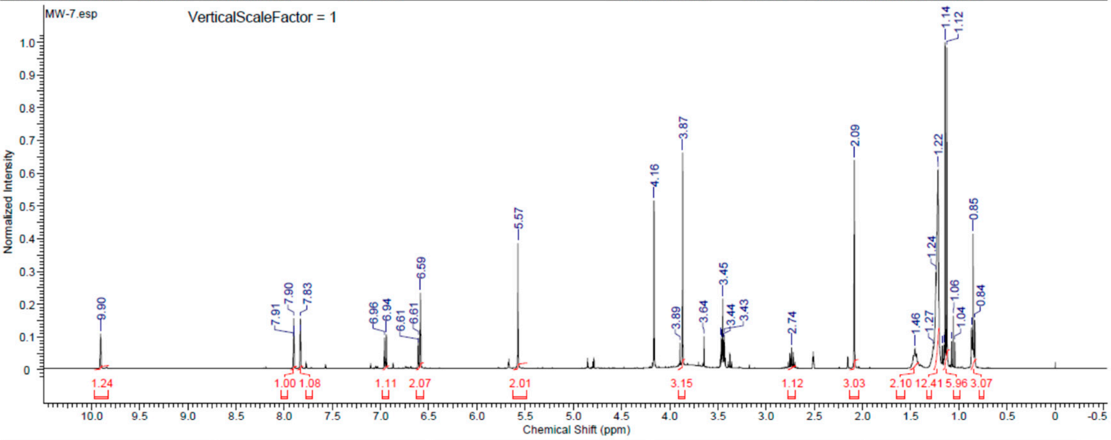

No.	(ppm)	(Hz)	Height	No.	(ppm)	(Hz)	Height	No.	(ppm)	(Hz)	Height	No.	(ppm)	(Hz)	Height	No.	(ppm)	(Hz)	Height
1	0.84	334.1	0.1511	9	1.15	461.3	0.0754	17	3.43	1373.1	0.1064	25	4.16	1664.7	0.5165	33	7.83	3131.8	0.1557
2	0.85	341.1	0.4144	10	1.17	468.2	0.0741	18	3.44	1376.8	0.0919	26	5.57	2229.5	0.3860	34	7.84	3133.6	0.1084
3	0.87	347.8	0.1293	11	1.22	486.6	0.6108	19	3.45	1379.6	0.2166	27	6.59	2634.2	0.2342	35	7.90	3157.8	0.1039
4	1.04	416.6	0.0844	12	1.24	495.9	0.2981	20	3.46	1383.7	0.0936	28	6.61	2642.0	0.0979	36	7.90	3159.6	0.1564
5	1.06	423.7	0.1646	13	1.27	506.9	0.0706	21	3.47	1386.3	0.0990	29	6.61	2643.6	0.0744	37	7.91	3161.3	0.0939
6	1.08	430.6	0.0864	14	1.46	582.4	0.0638	22	3.64	1457.7	0.1018	30	6.94	2775.5	0.1077	38	9.90	3960.9	0.1108
7	1.12	449.9	0.9842	15	2.09	833.9	0.6394	23	3.87	1546.3	0.6631	31	6.96	2783.0	0.0981				
8	1.14	456.8	1.0000	16	2.74	1094.1	0.0688	24	3.89	1556.8	0.0823	32	7.83	3130.3	0.0850				

This report was created by ACD/NMR Processor Academic Edition. For more information go to www.acdlabs.com/nmrproc/

2021-11-17 18:46:37

Acquisition Time (sec)	1.0000	*Date*	Nov 16 2021		*Date Stamp*	Nov 16 2021	
File Name	d:\Users\Michal\Desktop\MW-7-C13.fid\fid	*Frequency (MHz)*	100.57	*Nucleus*	13C	*Number of Transients*	1008
Original Points Count	22322	*Points Count* 32768	*Pulse Sequence* s2pul	*Receiver Gain*	56.00	*Solvent*	DMSO-d6
Spectrum Offset (Hz)	9724.6572	*Spectrum Type*	STANDARD	*Sweep Width (Hz)*	22321.43	*Temperature (degree C)*	25.000

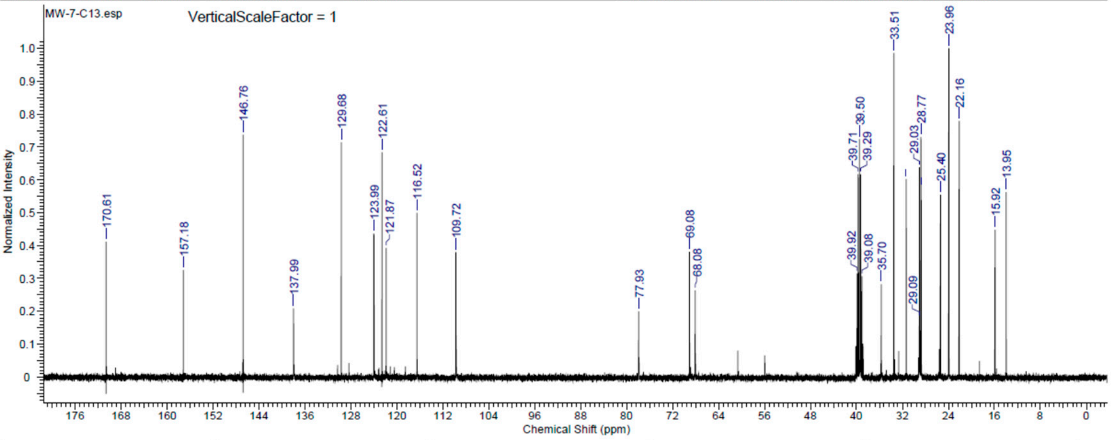

No.	(ppm)	(Hz)	Height	No.	(ppm)	(Hz)	Height	No.	(ppm)	(Hz)	Height	No.	(ppm)	(Hz)	Height	No.	(ppm)	(Hz)	Height
1	13.95	1403.3	0.5618	8	28.80	2896.5	0.5766	15	35.72	3592.7	0.2018	22	69.08	6947.0	0.3803	29	129.68	13041.8	0.7141
2	15.92	1600.8	0.4479	9	29.03	2919.0	0.6370	16	39.08	3930.6	0.3067	23	77.93	7836.7	0.1992	30	137.99	13877.0	0.2086
3	22.16	2228.9	0.7792	10	29.05	2921.7	0.5702	17	39.29	3951.0	0.6166	24	109.72	11033.6	0.3799	31	138.01	13879.1	0.1905
4	23.96	2409.4	1.0000	11	29.09	2925.1	0.1791	18	39.50	3972.1	0.7230	25	116.52	11717.6	0.4993	32	146.76	14759.2	0.7368
5	25.40	2554.5	0.5543	12	31.36	3153.3	0.6020	19	39.71	3993.2	0.6162	26	121.87	12256.4	0.3920	33	157.18	15806.9	0.3249
6	28.74	2890.4	0.5044	13	33.51	3369.9	0.9856	20	39.92	4014.4	0.3142	27	122.61	12330.0	0.6841	34	170.61	17157.8	0.4107
7	28.77	2893.8	0.7299	14	35.70	3590.6	0.2819	21	68.08	6846.2	0.2637	28	123.99	12469.6	0.4364				

1-methyl-3-octyloxymethylimidazolium vanillinoxyacetate (VA-8):

This report was created by ACD/NMR Processor Academic Edition. For more information go to www.acdlabs.com/nmrproc/

2021-11-19 17:11:30

Acquisition Time (sec)	3.9999	*Date*	Nov 17 2021	*Date Stamp*	Nov 17 2021		
File Name	d:\Users\Michal\Desktop\MW-8.fid\fid	*Frequency (MHz)*	399.91	*Nucleus*	1H	*Number of Transients*	64
Original Points Count	24752	*Points Count*	32768	*Pulse Sequence*	s2pul	*Receiver Gain*	20.00
Solvent	DMSO-d6	*Spectrum Offset (Hz)*	2746.2166	*Spectrum Type*	STANDARD	*Sweep Width (Hz)*	6188.12
Temperature (degree C)	25.000						

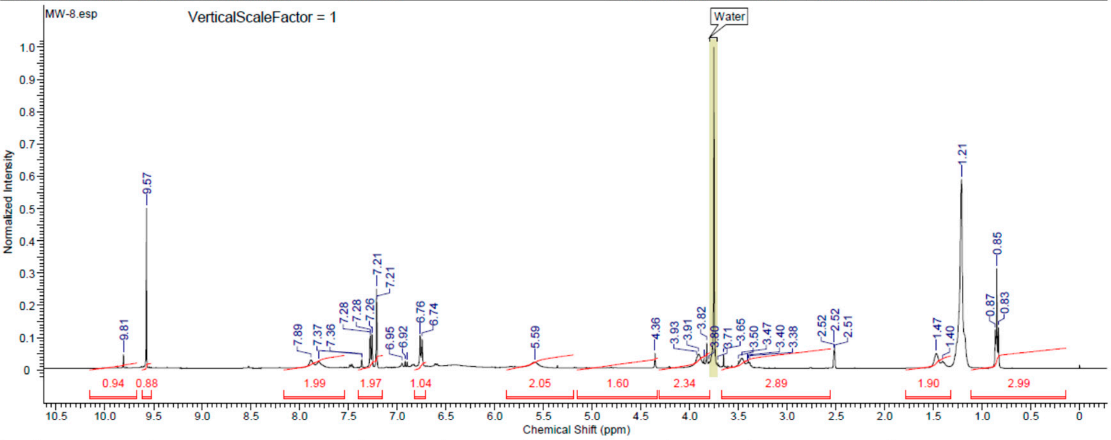

No.	(ppm)	Annotation	Layer No.	Created By	Created At	Modified By	Modified At
1	[3.72 .. 3.80]	Water	1	Michal	Pt 2021-11-19 15:40:36		

No.	(ppm)	(Hz)	Height
1	0.83	333.0	0.1307
2	0.85	340.0	0.3145
3	0.87	346.4	0.1240
4	1.21	484.4	0.5908
5	1.40	561.7	0.0259
6	1.47	587.9	0.0511
7	2.51	1004.5	0.0583
8	2.52	1006.2	0.0783
9	2.52	1008.1	0.0609
10	3.38	1353.0	0.0255
11	3.40	1359.4	0.0271
12	3.41	1364.9	0.0211
13	3.47	1385.8	0.0362
14	3.50	1399.4	0.0267
15	3.65	1460.2	0.0471
16	3.71	1485.2	0.0230
17	3.80	1520.1	0.0258
18	3.82	1529.5	0.0845
19	3.86	1541.8	0.0339
20	3.91	1563.5	0.0491
21	3.93	1570.9	0.0387
22	4.36	1741.6	0.0510
23	5.59	2236.4	0.0246
24	6.74	2697.2	0.0956
25	6.76	2705.3	0.1028
26	6.90	2759.0	0.0239
27	6.92	2767.3	0.0254
28	6.95	2780.1	0.0209
29	7.21	2882.9	0.2103
30	7.21	2884.7	0.2519
31	7.26	2901.7	0.1101
32	7.26	2903.6	0.1058
33	7.28	2909.9	0.1065
34	7.28	2911.7	0.0990
35	7.36	2944.0	0.0301
36	7.37	2945.7	0.0316
37	7.80	3121.2	0.0259
38	7.89	3153.3	0.0312
39	9.57	3828.4	0.5014
40	9.81	3922.7	0.0479

This report was created by ACD/NMR Processor Academic Edition. For more information go to www.acdlabs.com/nmrproc/

2021-11-19 17:11:05

Acquisition Time (sec)	1.1000	*Date*	Nov 17 2021	*Date Stamp*	Nov 17 2021		
File Name	d:\Users\Michal\Desktop\MW-8-C13.fid\fid	*Frequency (MHz)*	100.57	*Nucleus*	13C	*Number of Transients*	1056
Original Points Count	24554	*Points Count*	32768	*Pulse Sequence*	s2pul	*Receiver Gain*	60.00
Solvent	DMSO-d6	*Spectrum Offset (Hz)*	9725.3379	*Spectrum Type*	STANDARD	*Sweep Width (Hz)*	22321.43
Temperature (degree C)	25.000						

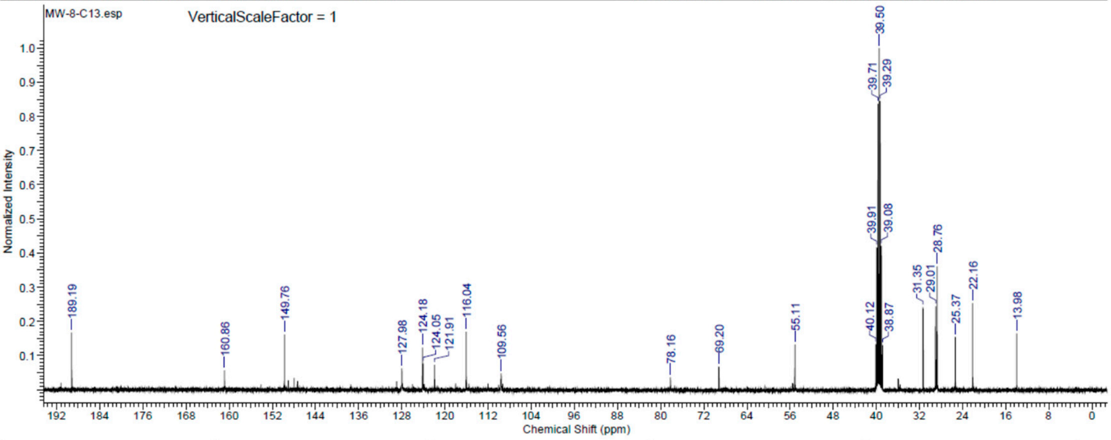

No.	(ppm)	(Hz)	Height
1	13.98	1406.0	0.1644
2	22.16	2228.2	0.2535
3	25.37	2551.8	0.1544
4	28.70	2886.3	0.1617
5	28.76	2892.4	0.3615
6	29.01	2917.6	0.2439
7	29.04	2920.3	0.1842
8	31.35	3152.6	0.2411
9	38.87	3908.8	0.1305
10	39.08	3929.9	0.4208
11	39.29	3951.0	0.8443
12	39.50	3972.1	1.0000
13	39.71	3993.2	0.8377
14	39.91	4013.7	0.4165
15	40.12	4034.8	0.1333
16	55.11	5542.3	0.1317
17	69.20	6959.3	0.0672
18	78.16	7860.5	0.0366
19	109.56	11017.9	0.0471
20	116.04	11669.9	0.1695
21	121.91	12259.8	0.0725
22	124.05	12475.1	0.0762
23	124.18	12488.0	0.1228
24	127.98	12870.9	0.0628
25	149.76	15060.3	0.1617
26	160.86	16177.5	0.0561
27	189.19	19026.3	0.1671

1-methyl-3-octyloxymethylimidazolium eugenoloxyacetate (EV-9):

This report was created by ACD/NMR Processor Academic Edition. For more information go to www.acdlabs.com/nmrproc/

2021-11-19 17:09:52

Acquisition Time (sec)	5.0001	*Date*	Nov 17 2021			*Date Stamp*	Nov 17 2021		
File Name	d:\Users\Michal\Desktop\MW-9.fid\fid			*Frequency (MHz)*	399.91	*Nucleus*	1H	*Number of Transients*	40
Original Points Count	30941	*Points Count*	32768	*Pulse Sequence*	s2pul	*Receiver Gain*	18.00	*Solvent*	DMSO-d6
Spectrum Offset (Hz)	2744.8352	*Spectrum Type*	STANDARD			*Sweep Width (Hz)*	6188.12	*Temperature (degree C)*	25.000

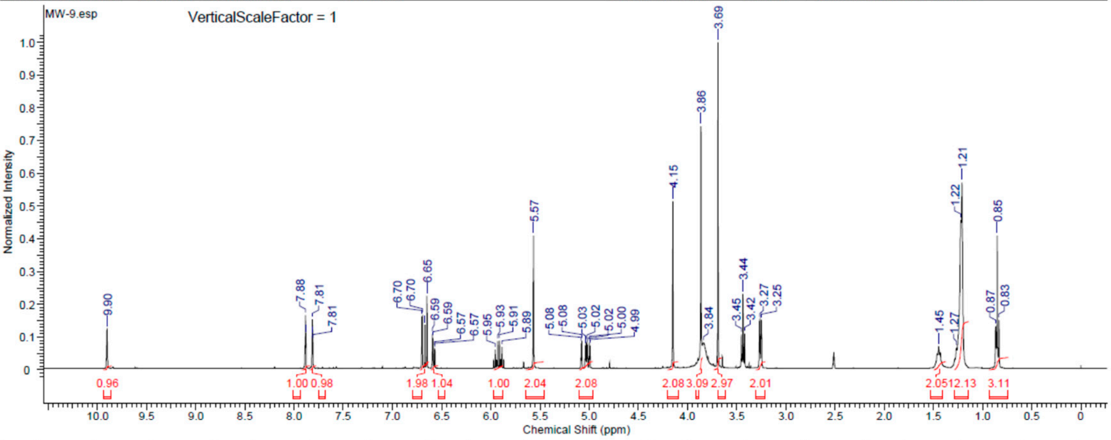

No.	(ppm)	(Hz)	Height	No.	(ppm)	(Hz)	Height	No.	(ppm)	(Hz)	Height	No.	(ppm)	(Hz)	Height	No.	(ppm)	(Hz)	Height
1	0.83	333.5	0.1522	11	3.44	1374.1	0.2322	21	5.02	2007.3	0.0712	31	5.95	2381.0	0.0616	41	7.81	3124.5	0.1536
2	0.85	340.5	0.4096	12	3.45	1380.7	0.1079	22	5.02	2008.2	0.0882	32	6.57	2626.5	0.0622	42	7.82	3126.0	0.1030
3	0.87	347.1	0.1334	13	3.69	1475.8	1.0000	23	5.03	2012.6	0.0838	33	6.57	2628.6	0.0668	43	7.88	3151.0	0.1073
4	1.21	483.4	0.5718	14	3.84	1534.8	0.0829	24	5.04	2014.8	0.0732	34	6.59	2634.8	0.0973	44	7.88	3152.7	0.1669
5	1.22	488.0	0.4570	15	3.86	1545.0	0.7433	25	5.08	2029.7	0.0884	35	6.59	2636.7	0.1086	45	7.89	3154.5	0.1011
6	1.27	506.3	0.0682	16	4.15	1659.2	0.5149	26	5.08	2031.8	0.0808	36	6.65	2658.6	0.2251	46	9.90	3959.8	0.1231
7	1.45	578.4	0.0711	17	4.99	1996.1	0.0736	27	5.57	2226.0	0.4095	37	6.67	2666.9	0.1364				
8	3.25	1299.5	0.1506	18	4.99	1997.3	0.0675	28	5.89	2354.0	0.0703	38	6.70	2677.9	0.1675				
9	3.27	1306.3	0.1533	19	5.00	1998.4	0.0838	29	5.91	2364.0	0.0891	39	6.70	2679.8	0.1622				
10	3.42	1367.4	0.1106	20	5.02	2006.1	0.0767	30	5.93	2371.0	0.0878	40	7.81	3122.8	0.0887				

This report was created by ACD/NMR Processor Academic Edition. For more information go to www.acdlabs.com/nmrproc/

2021-11-19 17:10:29

Acquisition Time (sec)	1.1000	*Date*	Nov 17 2021			*Date Stamp*	Nov 17 2021		
File Name	d:\Users\Michal\Desktop\MW-9-C13.fid\fid			*Frequency (MHz)*	100.57	*Nucleus*	13C	*Number of Transients*	688
Original Points Count	24554	*Points Count*	32768	*Pulse Sequence*	s2pul	*Receiver Gain*	56.00	*Solvent*	DMSO-d6
Spectrum Offset (Hz)	9724.6572	*Spectrum Type*	STANDARD			*Sweep Width (Hz)*	22321.43	*Temperature (degree C)*	25.000

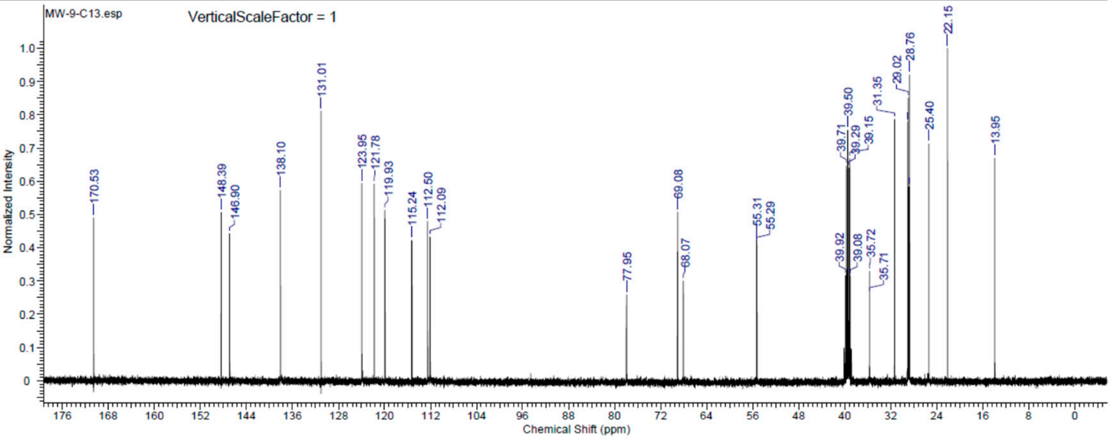

No.	(ppm)	(Hz)	Height	No.	(ppm)	(Hz)	Height	No.	(ppm)	(Hz)	Height	No.	(ppm)	(Hz)	Height	No.	(ppm)	(Hz)	Height
1	13.95	1403.3	0.6699	8	29.05	2921.0	0.7787	15	39.50	3972.1	0.7541	22	77.95	7838.7	0.2591	29	131.01	13175.4	0.8117
2	22.15	2227.5	1.0000	9	31.35	3152.6	0.7870	16	39.71	3993.2	0.6471	23	112.09	11272.7	0.4336	30	138.10	13888.6	0.5737
3	25.40	2554.5	0.7135	10	35.71	3591.3	0.2610	17	39.92	4014.4	0.3170	24	112.50	11313.6	0.4803	31	146.90	14773.5	0.4433
4	28.74	2890.4	0.6191	11	35.72	3592.7	0.3295	18	55.29	5560.7	0.4123	25	115.24	11589.5	0.4227	32	148.39	14922.7	0.5070
5	28.76	2892.4	0.9205	12	39.08	3930.6	0.3154	19	55.31	5562.1	0.4251	26	119.93	12060.9	0.5130	33	170.53	17149.6	0.4906
6	28.80	2896.5	0.5837	13	39.15	3936.7	0.6686	20	68.07	6845.5	0.3006	27	121.78	12246.9	0.5917				
7	29.02	2918.3	0.8508	14	39.29	3951.0	0.6408	21	69.08	6947.0	0.5084	28	123.95	12465.5	0.5945				

1-methyl-3-octyloxymethylimidazolium trimethylacetate (TR-10):

This report was created by ACD/NMR Processor Academic Edition. For more information go to www.acdlabs.com/nmrproc/

2021-11-17 18:45:29

Acquisition Time (sec)	4.9999	Date	Nov 17 2021			Date Stamp	Nov 17 2021
File Name	d:\Users\Michal\Desktop\MW-10.fid\fid	Frequency (MHz)	399.91	Nucleus	1H	Number of Transients	64
Original Points Count	32216	Points Count	32768	Pulse Sequence	s2pul	Receiver Gain	16.00
Solvent	DMSO-d6	Spectrum Offset (Hz)	2856.6477	Spectrum Type	STANDARD	Sweep Width (Hz)	6443.30
Temperature (degree C)	25.000						

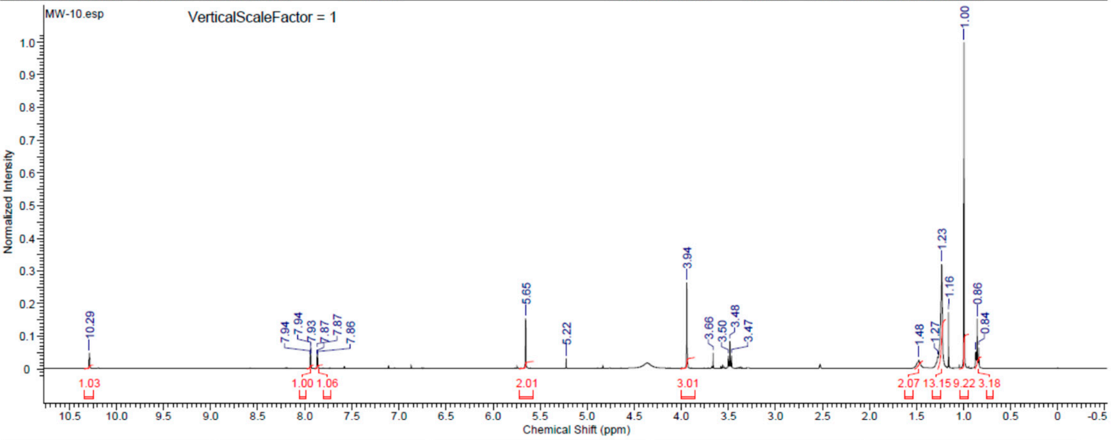

No.	(ppm)	(Hz)	Height
1	0.84	335.2	0.0656
2	0.86	342.5	0.1535
3	0.87	349.0	0.0520
4	1.00	398.2	1.0000
5	1.16	464.2	0.1733
6	1.23	492.7	0.3204
7	1.27	509.8	0.0368
8	1.48	590.3	0.0282
9	3.47	1386.3	0.0412
10	3.48	1393.0	0.0854
11	3.50	1399.4	0.0403
12	3.66	1463.9	0.0498
13	3.94	1576.0	0.2645
14	5.22	2088.3	0.0324
15	5.65	2260.7	0.1547
16	7.86	3144.0	0.0350
17	7.87	3145.8	0.0586
18	7.87	3147.4	0.0379
19	7.93	3173.1	0.0395
20	7.94	3174.7	0.0627
21	7.94	3176.5	0.0371
22	10.29	4114.3	0.0480

This report was created by ACD/NMR Processor Academic Edition. For more information go to www.acdlabs.com/nmrproc/

2021-11-17 18:47:04

Acquisition Time (sec)	1.1000	Date	Nov 17 2021			Date Stamp	Nov 17 2021
File Name	d:\Users\Michal\Desktop\MW-10-C13.fid\fid	Frequency (MHz)	100.57	Nucleus	13C	Number of Transients	784
Original Points Count	24554	Points Count	32768	Pulse Sequence	s2pul	Receiver Gain	56.00
Solvent	DMSO-d6	Spectrum Offset (Hz)	9726.3604	Spectrum Type	STANDARD	Sweep Width (Hz)	22321.43
Temperature (degree C)	25.000						

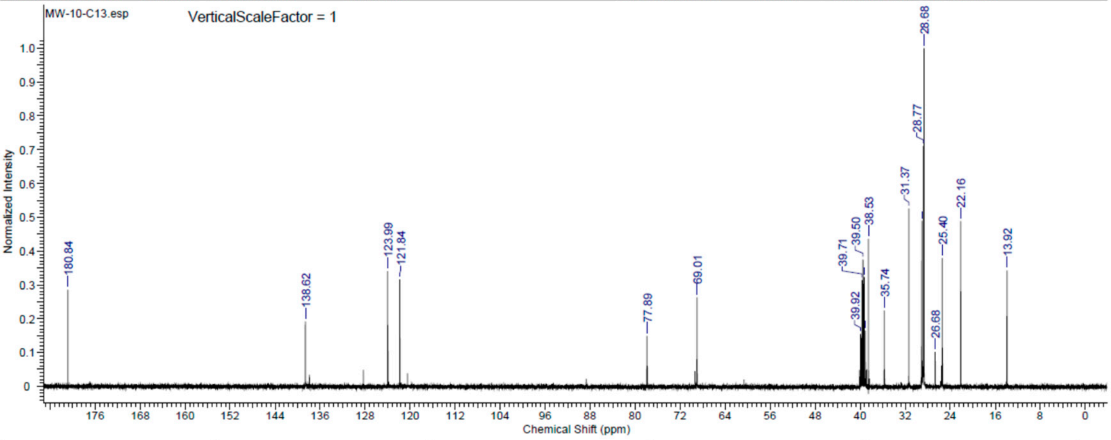

No.	(ppm)	(Hz)	Height
1	13.92	1400.2	0.3429
2	22.16	2228.5	0.4880
3	25.40	2554.9	0.3794
4	26.68	2682.9	0.1013
5	28.68	2883.9	1.0000
6	28.77	2893.4	0.7108
7	28.80	2896.1	0.4443
8	29.02	2918.6	0.4897
9	29.06	2922.7	0.4886
10	29.11	2927.5	0.0899
11	31.37	3154.3	0.5259
12	35.72	3592.3	0.1775
13	35.74	3594.4	0.2248
14	38.53	3874.4	0.4364
15	39.08	3930.2	0.1647
16	39.29	3951.3	0.3230
17	39.50	3972.5	0.3754
18	39.71	3993.6	0.3142
19	39.92	4014.7	0.1555
20	69.01	6939.8	0.2638
21	77.89	7832.9	0.1494
22	121.84	12253.3	0.3163
23	123.99	12469.3	0.3409
24	138.62	13940.7	0.1920
25	138.64	13942.8	0.1768
26	180.84	18186.1	0.2854
